# The CNS in inbred transgenic models of 4-repeat Tauopathy develops consistent tau seeding capacity yet focal and diverse patterns of protein deposition

**DOI:** 10.1186/s13024-017-0215-7

**Published:** 2017-10-04

**Authors:** Ghazaleh Eskandari-Sedighi, Nathalie Daude, Hristina Gapeshina, David W. Sanders, Razieh Kamali-Jamil, Jing Yang, Beipei Shi, Holger Wille, Bernardino Ghetti, Marc I. Diamond, Christopher Janus, David Westaway

**Affiliations:** 1 Centre for Prions and Protein Folding Diseases, University of Alberta, 204 Brain and Aging Research Building, Edmonton, AB T6G 2M8 Canada; 2 Department of Biochemistry, University of Alberta, Edmonton, AB Canada; 3 Center for Alzheimer’s and Neurodegenerative Diseases, UT Southwestern Medical Center, Dallas, USA; 4 Department of Pathology and Laboratory Medicine, Indiana University, Indianapolis, USA; 5 Center for Translational Research in Neurodegenerative Disease, University of Florida, Gainesville, FL USA

**Keywords:** Stochastic events, Aging, P301L mutation, Focal pathology, Neuronal loss, Transgenic mouse

## Abstract

**Background:**

*MAPT* mutations cause neurodegenerative diseases such as frontotemporal dementia but, strikingly, patients with the same mutation may have different clinical phenotypes.

**Methods:**

Given heterogeneities observed in a transgenic (Tg) mouse line expressing low levels of human (2 N, 4R) P301L Tau, we backcrossed founder stocks of mice to C57BL/6Tac, 129/SvEvTac and FVB/NJ inbred backgrounds to discern the role of genetic versus environmental effects on disease-related phenotypes.

**Results:**

Three inbred derivatives of a TgTau^P301L^ founder line had similar quality and steady-state quantity of Tau production, accumulation of abnormally phosphorylated 64–68 kDa Tau species from 90 days of age onwards and neuronal loss in aged Tg mice. Variegation was not seen in the pattern of transgene expression and seeding properties in a fluorescence-based cellular assay indicated a single “strain” of misfolded Tau. However, in other regards, the aged Tg mice were heterogeneous; there was incomplete penetrance for Tau deposition despite maintained transgene expression in aged animals and, for animals with Tau deposits, distinctions were noted even within each subline. Three classes of rostral deposition in the cortex, hippocampus and striatum accounted for 75% of pathology-positive mice yet the mean ages of mice scored as class I, II or III were not significantly different and, hence, did not fit with a predictable progression from one class to another defined by chronological age. Two other patterns of Tau deposition designated as classes IV and V, occurred in caudal structures. Other pathology-positive Tg mice of similar age not falling within classes I-V presented with focal accumulations in additional caudal neuroanatomical areas including the *locus coeruleus.* Electron microscopy revealed that brains of Classes I, II and IV animals all exhibit straight filaments, but with coiled filaments and occasional twisted filaments apparent in Class I. Most strikingly, Class I, II and IV animals presented with distinct western blot signatures after trypsin digestion of sarkosyl-insoluble Tau.

**Conclusions:**

Qualitative variations in the neuroanatomy of Tau deposition in genetically constrained slow models of primary Tauopathy establish that non-synchronous, focal events contribute to the pathogenic process. Phenotypic diversity in these models suggests a potential parallel to the phenotypic variation seen in P301L patients.

**Electronic supplementary material:**

The online version of this article (10.1186/s13024-017-0215-7) contains supplementary material, which is available to authorized users.

## Background

Major questions in neurodegenerative disease research include the origins of sporadic disease, the time-lag before germline mutations in central nervous system (CNS)-expressed genes produce neurological symptoms and the degree of impact of prion-like effects upon CNS dysfunction. All of these issues are germane to diagnostic and therapeutic strategies and all occur in the literature of diseases involving the Tau protein. Primary Tauopathies refer to syndromes with aberrant forms of Tau, a microtubule-associated cytoplasmic protein. While primary Tauopathies include corticobasal degeneration and progressive supranuclear palsy, cases with germline mutations in the Tau gene (*MAPT*) are also notable. These were originally detected in the context of a clinical syndrome referred to as FTDP-17 (Frontotemporal Dementia with Parkinsonism linked to chromosome 17) [1]. These *MAPT* mutations included missense mutations in the coding sequence or intronic alterations that shifted the WT steady-state ratio of 3-repeat to 4-repeat spliced forms to lead to more 4R Tau [2, 3]. The term “Tauopathy” has been used as a descriptor for any neurologic syndrome that feature abnormal forms of this protein; for example, Alzheimer’s disease (AD) can be considered a secondary Tauopathy where neurofibrillary tangles (NFTs) containing hyperphosphorylated forms of Tau are a hallmark feature, yet where *MAPT* is free of mutations that alter protein structure or spliced forms.

Animal models based on mutated proteins can guide our understanding of pathogenic events and mutant forms of Tau have already been used for over 15 years to create rodent models of neurodegenerative disease [4, 5]. These mice have pathologies that recapitulate those seen in human disease and have been used extensively in preliminary tests of therapies [6–8]. With regards to accuracy in modeling of pathophysiological processes, most neurodegenerative diseases - and specifically frontotemporal dementia (FTD) are not congenital and instead occur with onset in mid to late life. On the other hand, overexpression of transgenes, while convenient to attain fast onset of pathology and shortened durations for experimental studies, can lead to changes in the CNS of adolescents rather than individuals with aged nervous and immune systems. The use of low-expressor mice has been applied to Aβ-related pathologies [9, 10] and is also of interest in the context of Tau [11]. In this vein, we created transgenic (Tg) mice expressing a P301L mutant version of the longest spliced form of human Tau, 2 N, 4R [“TgTau(P301L)23,027 mice”] at low levels (1.7× endogenous full-length Tau [12])]. Because the transgene construct is based on a cDNA clone and lacks introns between protein coding exons, simultaneous production of human 3R and 4R Tau mRNA is eliminated as an operational variable in this model. Denoted for brevity as TgTau^P301L^, these mice develop, albeit slowly, pre-tangles and also a number of florid pathologies including numerous granofibrillary tangles (“GFTs”, used to describe accumulations of Tau in astrocytes) and NFT-like structures in the CNS. Besides immunohistochemistry, Tau inclusions in these mice were visualized with Thioflavin S, Gallyas-Braak and Bielschowsky staining procedures [12]. Notably, motor defects present in some other Tau Tg mice (due to extensive Tau deposition in the spinal cord) are absent in our model. Cell death was noted in TgTau^P301L^ mice in the temporal and hippocampal formations thus paralleling cell death widely reported amongst TgTau models [13].

Interestingly, in the original description of this TgTau^P301L^ transgenic line, the time of onset of clinical disease differed between Tg colonies derived from the same founder stock yet maintained at different laboratory sites (Okayama and Toronto). Moreover, heterogeneity also existed within cohorts of age-matched Tg mice of the same colony with regards to levels of insoluble Tau and memory function [12]. Given that phenotypic variation occurs in patients carrying the same P301L mutation [1, 14–16], we extrapolated that the TgTau^P301L^ line might be manifesting a related biological effect and, ultimately, might allow us to understand parameters that dictate phenotypic diversity [17].

**Fig. 1:**
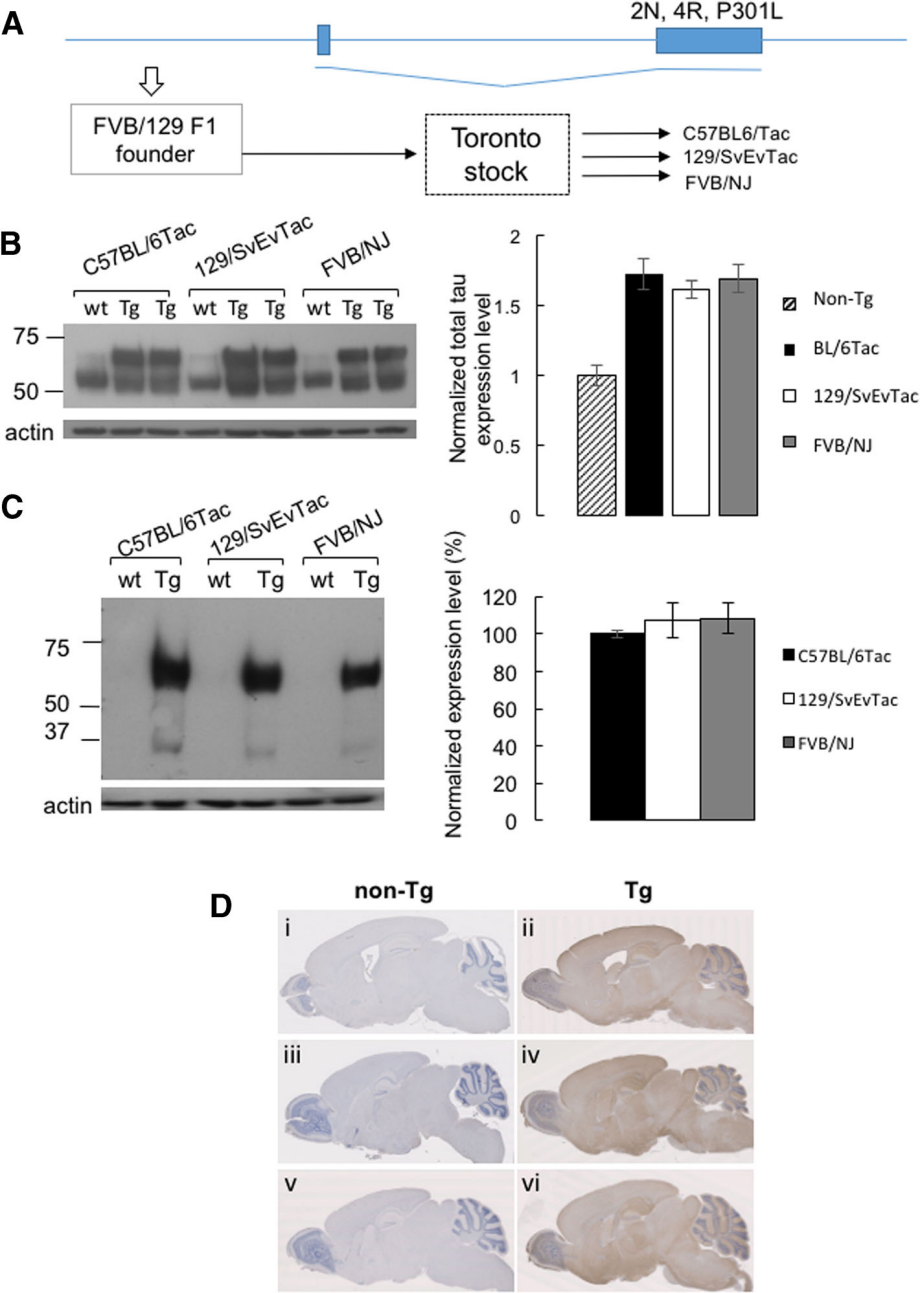
Total human Tau and total phosphorylated Tau in 3 congenic strains of TgTau^P301L^ mice. **a** schematic of the cosmid transgene and derivation of a founder Tg line (open arrow). A Toronto breeding stock (T, indicated by dashed square) was used to generate three inbred derivatives. In panels b and c, brain homogenates of Tg mice from all 3 congenic strains and controls were analyzed for total Tau and total human Tau expression levels using western blot. **b** A representative blot showing total Tau expression in Tg mice of all congenic strains and control animals. Graph represents relative expression levels of total Tau normalized to actin loading control and then compared to control non-Tg mice *n* = 2 per Tg sample per inbred stain with values for non-Tg sample pooled from the three strains and assigned the value “1.0”. Antibody is DA9:1/500). TgTau^P301L^ mice have ~1.7X Tau expression level (1.77, 1.61, 1.69 for C57BL/6Tac, 129/SvEvTac and FVB/NJ respectively) compared to controls. **c** A representative blot showing total human Tau expression level in Tg mice and controls. Graph represents total human Tau expression levels in transgenic mice of all 3 congenic strains (*n* = 3 each) normalized to C57BL/6Tac (100%; antibody CP27, 1/500). **d** Total human Tau distribution (CP27) in brain of Tg mice (280–324 days old) from C57BL/6Tac (ii), 129/SvEvTac (iv) and FVB/NJ (vi) backgrounds, with corresponding non-Tg animals shown in panels i, ii and iii

In studies presented here we have considered two alternative hypotheses to account for this effect. First, that phenotypic heterogeneity reflects the action of genetic modifier loci and can be exacerbated by moving the transgene onto different inbred backgrounds (Fig. 1a). Second, that phenotypic variations in TgTau^P301L^ mice do not reflect the genetic modifier loci but rather reflect stochastic cell biological events and/or environmental inputs. To explore these two hypotheses, we imposed constraints on the original transgenic system wherein the TgTau^P301L(T)^ founder stock was used to create three inbred derivatives. Our studies defined variations in Tau deposition shared between (and independent of) the three inbred genetic backgrounds, thus suggesting the action of extrinsic disease modifiers to trigger stochastic events. Moreover, different biochemical signatures associated with different pathologies strongly imply that these inbred animal models can generate distinct Tau strains.

## Methods

### Animals

TgTau^P301L^ mice and their non-Tg littermates have been previously derived by injections into oocytes from 129/SvEvTac x FVB/J F_1_ mice and the Tg offspring bred to 129/SvEvTac mice to enrich for this genetic background. For the studies here, starting with mice sourced from the University of Toronto, back-crosses were continued with 129/SvEvTac mice (N11) to make a congenic derivative [18, 19]. Crosses were also carried out to make an FVB/J congenic derivative (N12). Lastly, we attempted to make a fully congenic derivative using C57BL/6Tac mice but, due to lower fecundity encountered in this background, we achieved an enriched stock after N8 backcrosses. Animals were maintained in ventilated racks (Tecniplast, Green Line) and fed irradiated chow (LabDiets, 5053). They were housed with a 12 h/12 h light/dark cycle. Cage environmental enrichment comprised 5 cm diameter plastic tubes and nesting material (“Nestlets”, Ancare Inc.). All animal experiments were performed in accordance with local and CCAC guidelines.

For the collection of brains for analysis by the isotropic fractionator technique, animals were anesthetized by isoflurane inhalation, perfused with 25 mL phosphate saline then 25 mL 4% phosphate-buffered paraformaldehyde (PFA) both at a flow rate of 4–5 mL/min. For western blot experiments, mice were sacrificed by cervical dislocation to exclude post-mortem phosphorylation artefacts due to anesthesia-induced temperature changes [20]. Tissue was immediately extracted, frozen on dry ice and kept at −80 °C until use.

### Genotyping and genome-wide analysis of SNPs

For routine genotyping of litters, tail-derived genomic DNA was amplified with primers 1572 = 5′. TGGATCTTAGCAACGTCCAGTCC.3′ and Primer 1587 = 5′. CTCTCCTCTCCACAATTATTGACCG.3′. PCR cycle conditions were 94 °C 3 min (94 °C 20 s, 55 °C 20 s, 72 °C 30 s) ×35, 72 °C 7 min, 4 °C hold, yielding a diagnostic fragment of 521 bp. For genome-wide analysis of single nucleotide polymorphisms (SNPs), genomic DNA from brain of representative Tg animals of the three backgrounds (backcross numbers indicated as above) were genotyped at 384 dimorphic SNP loci positioned, on average, every 7 Mbp across the mouse genome (Charles River Genome Testing Services, Wilmington, MA). Results are reported as overall divergence and divergence in heterozygous or homozygous state from inbred strain reference standards.

### Evaluation of disease-related symptoms in aged mice

To assess clinical manifestation of disease, we produced initial cohorts of 58, 79 and 53 transgenic animals and their littermates (C57BL/6Tac, 129/SvEvTac and FVB/NJ backgrounds, respectively) for aging. In routine health checks, we scored the cohorts with observation periods of up to 750 days of age for kyphosis, tremors, hypokinesia and tail rigidity (present/absent). We then recorded the number of animals manifesting at least three of these symptoms simultaneously on three consecutive days; in the case of weight loss of 20% or more the animals were euthanized. The development of symptoms in Tg animals was irreversible. Non-transgenic littermates were also retained for aging analysis (*n* = 23, 21 and 23 C57BL/6Tac, 129/SvEvTac and FVB/NJ backgrounds, respectively).

### Quantification of phosphorylated and total tau protein

Brain samples were homogenized 10% *w*/*v* in 2% sarkosyl phosphate-buffered saline containing 1% protease inhibitor cocktail (Sigma, St. Louis, MO). Total protein concentration in samples was determined by Bicinchoninic Acid assay (Pierce). Twenty μg total protein from each sample was loaded and electrophoresed on a 10% Tris-tricine SDS-PAGE gel. Western blotting was then performed on samples as described below.

### Extraction of phosphorylated tau protein

Fractions of brain were prepared as previously described [21]. Briefly, tissues were homogenized in 10 volumes of Tris-buffered saline [TBS: 50 mM Tris/HCl (pH 7.4), 274 mM NaCl, 5 mM KCl, 1% protease inhibitor mixture, 1% phosphatase inhibitor cocktail (Sigma) and 1 mM phenylmethylsulfonyl fluoride (PMSF)]. The homogenates were then centrifuged at 27,000×*g* for 20 min at 4 °C to obtain supernatant (“SUP1”) and pellet fractions. The pellet was then re-suspended in five volumes of high salt/sucrose buffer [0.8 M NaCl, 10% sucrose, 10 mM Tris/HCl, (pH 7.4), 1 mM EGTA, and 1 mM PMSF] and centrifuged at 27,000×*g* for 20 min at 4 °C. The supernatants obtained from this step were collected and incubated with sarkosyl (1% final concentration; Sigma) for one hour at 37 °C, followed by centrifugation at 150,000×*g* for one hour at 4 °C to obtain salt and sarkosyl-extractable (“SUP3”) and sarkosyl-insoluble (“P3”) fractions. The P3 pellet was re-suspended in TE buffer [10 mM Tris/HCl (pH 8.0), 1 mM EDTA] to a volume equivalent to half of that of the brain specimens used to produce brain homogenates. To prepare an S1P fraction containing soluble Tau oligomers, a portion of the SUP1 fraction was further centrifuged at 150,000 x*g* for 20 min.

### Western blots

Western blotting was performed as described previously [12, 22]. Samples were prepared in loading buffer containing SDS and 2-mercaptoethanol and boiled for 10 min. They were then electrophoresed on 10% Tris-tricine gels using a BioRad system and transferred to polyvinyl difluoride (PVDF; Millipore) membranes (wet transfer) and blots were then blocked with 5% skim milk in 1xTBS-0.1% Tween 20 for one hour at room temperature and incubated with primary antibodies at 4 °C overnight. CP13 (detecting phosphorylated Ser202), CP27 (detecting total human Tau protein) and PHF1 (detecting phosphorylated S396 and Serine 404) antibodies (kind gifts from Dr. Peter Davies, Albert Einstein College of Medicine) were used at 1/500 dilution, while ET3 (detecting 4R Tau residues 273–288) from the same source was used at 1/250 dilution. Membranes were subsequently incubated with secondary antibody (BioRad) at 1/10000 for one hour at room temperature and visualized using enhanced chemiluminescence (ECL, Pierce). Anti-actin antibody (Sigma) was used for quantification blots (1/2000 dilution).

### Trypsin digestion of sarkosyl-insoluble tau extracts

Sarkosyl insoluble fractions (P3) were subjected to trypsin digestion as previously described [23, 24] with some modifications. Briefly, reactions were set up using 10 μg of P3 fraction, or 15 μg for Class IV animals with 1/100 part sequencing grade trypsin (Pierce). After 30 min of incubation at 37 °C, the reaction was stopped by addition of sample buffer and boiling for 10 min. Samples were then resolved on NuPAGE Novex 4–12% gradient gels and prepared for immunoblotting. ET3 antibody was used for detection of Tau fragments.

### Isotropic fractionator

The isotropic fractionator method for quantifying neuronal loss in tissue samples was performed as previously described with minor modifications [25, 26]. Briefly, perfused and post-fixed brain samples were mechanically dissociated and homogenized in 10 volumes of a solution of 40 mM sodium citrate and 1% Triton X-100. The homogenates were collected and the homogenizer washed at least twice to collect any residual cells. To visualize nuclei and obtain total cell counts, 20 μL of 100 μg/mL stock solution of 4′,6-diamidino-2-phenylindole (DAPI, Sigma) was added to the cell suspension and the total number of cells were counted (final concentration of DAPI was 0.20 μg/ml). Counting was done in a semi-automated manner: pictures of the quadrants were taken (by a QImaging retiga 2000 camera) and a sub-routine software, adjusted to detect fluorescent signals from stained nuclei (InCell Analyzer software, GE-Healthcare), was used for unbiased counting of the cells. All four quadrants in both the upper and lower grids of the hemocytometer were used for counting and the results were averaged. To determine total neuron counts, 1 mL of each cell suspension was removed, washed with PBS, and centrifuged for 10 min at 5000 *xg*. Cells were then incubated in anti-NeuN antibody (1:200; Millipore) for two hours followed by incubation in a secondary anti-mouse IgG-Alexa-Fluor 594 for one hour (1:200; Millipore). Cells were then counted with a hemocytometer using a Nikon eclipse 90*i* microscope and the percentage of NeuN-stained nuclei was recorded. Total cell numbers and neuronal numbers were then calculated by multiplying the number of cells/mL by the final volume.

### Immunohistochemistry

Each specimen was fixed by immersion in neutral-buffered 10% formalin. Samples were subsequently dehydrated and paraffin-embedded. 6 μm sagittal sections were rehydrated and endogenous peroxidase activity was blocked by treatment with 3% hydrogen peroxide for six minutes. After washes, sections were incubated overnight with primary antibody anti-Tau (1:200; ThermoFisher), AT8 (1:200, Thermofisher), MC1 (1:100, Peter Davies), CP27, RZ3 and PHF1 (all at 1:200, Peter Davies), visualized with horseradish peroxidase using the DAKO ARK™ kit according to the manufacturer’s instructions. Sections were counterstained using Mayer’s hematoxylin, dehydrated and cover-slipped with permanent mounting medium. For Thioflavin-S-staining, following de-paraffination and rehydration, slices were immersed in 0.3% potassium permanganate for five minutes, and the reaction was quenched in a solution containing 1% potassium metabisulfite and 1% oxalic acid. Slices were then immersed in a 0.05% Thioflavin S solution for 30 min. Then the slides were differentiated in 80% alcohol, incubated in phosphate buffer for 30 min and counterstained with propidium iodine before mounting.

For classification of pathology types, AT8-immunostained sagittal sections 0.72 mm lateral to the midline were assessed: a scheme was devised based upon recurring patterns of deposition occurring in 113 brains from aged Tg animals. This scheme was then formalized (See Fig. 5) and then brains were inspected by one or two further observers blind to the original designations to confirm classification of a given sample. A fraction of the brain samples did not achieve a consensus classification (“other”) because of the coincident appearance of two or more patterns, or because of different morphologies or because there was no AT8 staining.

### Homogenate and lysate preparation

Hemi-sected transgenic mouse brains were stored at −80 °C prior to homogenization. Frozen brains were placed onto a chilled metal block on dry ice and were transected coronally at the level of the midbrain to separate caudal and rostral brain sections (see also Fig. 15). Sections were placed in 1 mL PBS prepared with protease inhibitor tablet (Roche) and were sonicated using a probe sonicator (Omni Sonic Ruptor 400) at 30% pulse/30% power, 20 times, 10-s cycles. The resulting homogenates were centrifuged at 10,000 *xg* for 15 min at 4 °C. and supernatants used for subsequent steps [27]. Clone 1, Clone 9, and Clone 10 lysates were prepared as described previously [27]. Cell pellets were lysed in 0.1% Triton-X/PBS solution plus protease inhibitor cocktail (“Complete”, Roche) and were clarified with sequential five-minute 500 *xg* and 1000 *xg* spins. Protein concentrations in brain homogenates and cell lysates were measured by Bradford assay (Bio-Rad) and were normalized to 5 μg/μL and stored at −80 °C until use.

### Negative stain electron microscopy

Aliquots (5 μL) of sarkosyl-insoluble P3 fractions were loaded onto freshly glow-discharged 400 mesh carbon coated copper grids (Electron Microscopy Sciences) and adsorbed for ~1 min. Next, the grids were washed with 50 μL each of 0.1 M and 0.01 M ammonium acetate respectively and negatively stained with 2 × 50 μL of freshly filtered 2% uranyl acetate. After drying, the grids were examined with a Tecnai G20 transmission electron microscope (FEI Company) using an acceleration voltage of 200 kV. Electron micrographs were recorded with an Eagle 4 k × 4 k CCD camera (FEI Company). The morphology of individual Tau filaments was readily visible and classified into “straight filaments”, “coiled filaments”, and “twisted ribbon-like filaments”.

### Cell culture

Clone 1 cells (monoclonal HEK293 cells stably expressing Tau RD P301L/V337 M-YFP, described previously [27]), were cultured in Dulbecco’s modified Eagle’s medium (Gibco) supplemented with 10% fetal bovine serum (HyClone), 1% penicillin/streptomycin (Gibco), and 1% Glutamax (Gibco). Cells were maintained and passaged in 10 cm dishes at 37 °C, 5% CO_2_, in a humidified incubator.

### Transduction of homogenates into clone 1 cells

Clone 1 cells were plated at 100,000 cells/well in 24-well plates. 18 h later, cells were transduced with brain homogenates or cell lysates packaged into liposomes. Liposomes were prepared as follows: 100 μg of clarified homogenate was combined with OptiMEM (Gibco) to a final volume of 50 μL. 48 μL OptiMEM and 2 μL lipofectamine-2000 (Invitrogen) were then added to the OptiMEM/lysate mixture to a final volume of 100 μL. After a 15-min incubation, liposome preparations were added to the cells. 18 h later, cells were washed, trypsinized, and re-plated into 6-well plates. At confluency (96 h following the original lysate transduction), cells were passaged onto coverslips in 24-well plates. 120 h following transduction, cells were fixed with 3% PFA, washed once with PBS and stained with DAPI (1:1000 diluted from 1 mg/mL stock) in 0.1% Triton-X/PBS for 10 min. DAPI solution was replaced with PBS and coverslips were mounted using ProLong Gold Antifade Reagent (Life Technologies) and sealed with nail polish. Prior to imaging, mounted coverslips were stored at 4 °C.

### Confocal analysis of seeded clone 1 cells

Confocal microscopy was performed using a Zeiss LSM 780 PASCAL system coupled to a Zeiss Axiovert 200 M microscope. A pinhole size of 0.8 μm was used for the collection of all images. The imager was blinded to the identity of the samples to avoid bias in selection of representative fields of cells.

### Data analysis

Departures from normal distribution were checked using the Kolmogorov-Smirnov (K-S) goodness of fit test. A general linear model of factorial ANOVA (Statistical Package for Social Sciences, SPSS v.22, Inc. Chicago), with genotype, line/genetic background as between subject factors, or repeated measures analysis of variance (RMANOVA) with the type of cell count (total cell and neuronal counts) as within subject factors were used to analyze the data. Eta squared (η^2^) was used to estimate the effect size, i.e. the proportion of variance associated with each of the main effects and interactions. Bonferroni adjustment of α level (MODLSD Bonferroni t-tests, SPSS v22) was applied in multiple planned comparisons. In the case when data represented discrete category measures on a nominal scale and did not meet the assumption of parametric statistics, a χ2 test of independence was used to test for homogeneity between the groups.

## Results

### Genetic profiles and tau expression in sublines of TgTau^P301L^ mice

After a series of backcrosses of a TgTauP301L founder stock (Fig. 1a) genomic DNA from an individual of each inbred derivative sub-line (C57BL/6Tac, 129/SvEvTac or FVB/NJ) was analyzed in a genome-wide 384 single nucleotide polymorphism (SNP) array versus reference standards. For the tested samples, the “call rate” for scoring polymorphisms on the array was between 98.2% and 99.7% (Additional file 1: Table S1) with the positions of the residual unscored SNPs not being shared by the three inbred derivatives. For called loci each Tg subline yielded 99.2% or higher values for being a homozygous or heterozygous match to SNPs in the reference genome (Additional file 1: Table S1); these data indicate incipient congenic status for the 129/SvEvTac and C57BL/6Tac sublines and congenic status for the FVB/NJ sub-line (99.87% match, 99.9% desired). For 21 scored variations from the inbred strain reference standards, only two SNP variants were shared by different backgrounds: (i) VF at position Chr01–26 in Tg mice versus VV expected in the 129/SvEvTac and FVB reference samples (where V and F refer to the two fluorescent dyes used for tagging allele-specific oligonucleotides) and (ii) VF in Tg mice instead of FF at position Chr07–05 in 129/SvEvTac and C57BL/6 versus reference samples. Overall, these data confirm matching between the three inbred Tg sublines with respect to reference genomic DNAs and that residual distinctions are dispersed across the mouse genome in chromosomes 1, 5, 7 and 14.

The parental Tg line was notable for a low level of transgene expression (previously estimated at 1.7× endogenous); to verify that this parameter was unaltered by the series of back-crosses, we quantitated western blot analyses on brain material of young, 90-day old mice from the three inbred sublines, to measure levels of total tau (i.e., endogenous tau plus human tau) (Fig. 1b). Considering transgene-encoded tau measured with an anti-human tau CP27 antibody, we did not detect significant differences in steady-state expression levels (Fig. 1c) between the three genetic derivatives. While histological analysis with CP27 antibody specific for human tau was not above background for non-Tg mice (as anticipated), similar patterns of dispersed immunoreactivity were present in each of the congenic or incipient congenic derivatives (Fig. 1d). These data are compatible with the broad pattern of neuronal expression associated with the parental Syrian hamster prion protein gene [28] and seen in other Tg lines made with the cos.Tet expression vector derived from this gene [29, 30].

**Fig. 2:**
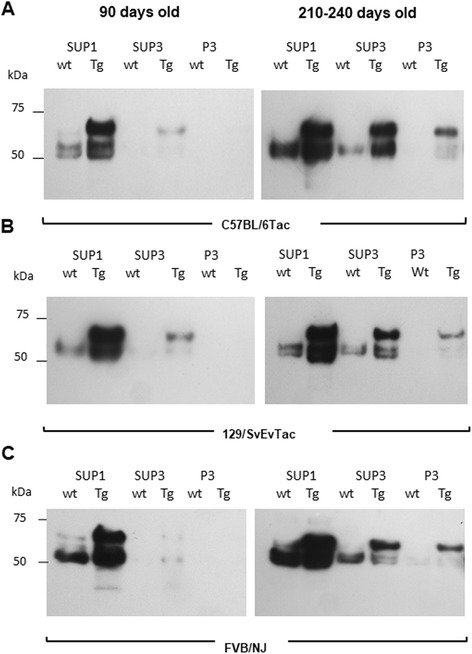
Presence of phosphorylated Tau species in fractioned brain of 90 and 240 days old mice from 3 genetic backgrounds. Half brains of TgTau^P301L^ and non-Tg littermate controls were subjected to fractionation and presence of phosphorylated Tau species were investigated in different fractions. Inbred strains were investigated at 90 and 210–240 days of age, as indicated. **a** C57BL/6Tac, (**b**) 129/SvEvTac, and (**c**) FVB/NJ. For all samples, 10 μg of total protein was loaded on the gel. Antibody: CP13 (1/500; phosphoserine 202). SUP = Supernatant and P = pellet fractions

We next analyzed fractionated brain samples in all three genetic backgrounds to determine relative levels of hyperphosphorylated Tau species (64–68 kDa) in a standard procedure to yield TBS-extractable (SUP1), salt and sarkosyl-extractable (SUP3) and sarkosyl-insoluble (P3) fractions. Figure 2 shows western blots of fractionated brains of Tg mice at 90 and 240 days of age versus non-Tg littermates (“WT”). Using the CP13 antibody, an antibody that also detects endogenous mouse Tau species, at 90 days, the P3 fraction was not populated with phosphorylated Tau species in any of the three genetic backgrounds. This situation altered at a 240-day time point wherein the human transgene encoded species became visible in the P3 fraction of each subline. Thus, an accumulation of insoluble Tau species was apparent comparing 90 and 240-day time-points in asymptomatic mice of all three genetic backgrounds.

### Neurologic symptoms and tau species in aged Tg mice

Tg mice were scrutinized for neurological signs of disease as they approached and entered their second year of life, with the presence of three simultaneous signs of disease required for a score. Using initial cohorts of 58, 79 and 53 transgenic animals (C57BL/6Tac, 129/SvEvTac and FVB/J backgrounds, respectively) surviving over 240 days and observation periods of up to 750 days, all three genetic backgrounds presented with signs of neurologic disease (Additional file 2: Figure S1), albeit with incomplete penetrance (34.4%, 20.2% and 28.3%, respectively). Symptomatic presentation occurred in a different manner from progressive age-related losses scored in non-Tg littermates (and documented from large-scale breeding studies of inbred strains [31]). Mean elapsed times for onset of symptoms (days) ± SD corresponded to 543.8 ± 123.4, 582.4 ± 74.6 and 509.8 ± 96 for Tg lines maintained on C57BL/6Tac, 129/SvEvTac and FVB/NJ genetic backgrounds and were not different between groups (χ^2^(2) = 5.5, *p* = 0.06.

### Quantification of neuronal loss in aged TgTau^P301L^ mice

**Fig. 3:**
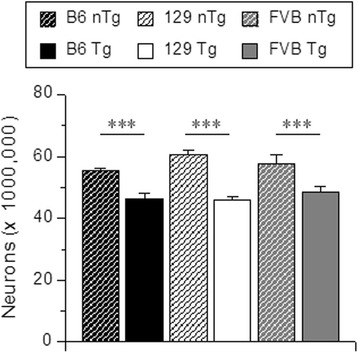
Quantification of neurons in whole brain of aged TgTau^P301L^ and non-Tg littermate mice. Total number of neurons in TgTau^P301L^ and non-transgenic mice. *** *p* < 0.001. *n* = 7 for all genotypes

Earlier characterization of TgTau^P301L^ mice in the Okayama cohort demonstrated brain atrophy in 540 days old animals, loss of CA1 and CA2 neurons and thinning of the CA3 cell layer [12]. We investigated this issue in the newer iterations of this model using the isotropic fractionator technique [32], a quantitative method reported as having similar accuracy to unbiased stereology [32, 33]. We used a total of 42 aged mice from all three congenic lines for this purpose: 14 of FVB/NJ line (7 nTg (4♂/3♀) and 7 Tg (3♂/4♀)), 14 of 129SvEv (7 nTg (3♂/4♀) and 7 Tg (5♂/2♀)), and 14 of C57BL/6 line (7 nTg (4♂/3♀) and 7 Tg (5♂/2♀)). The average age was 547.8 ± 13.2, 711.4 ± 5.7, and 658.7 ± 15.1 days for the FVB/NJ, 129/SvEvTac, and C57BL/6Tac cohorts, respectively. The distribution of age ranges of 129 and B6 lines was normal, but it was negatively skewed towards older age range (skewness = −0.243) for the FVB line (D(14) = 0.243, *p* < 0.05, K-S one-sample test). The comparison of the age between the strains (genetic background), genotype and sex revealed significant strain effect (F(2,30) = 40,8, *p* < 0.001) with no other factors and interactions being significant at α = 0.05. Post hoc comparisons revealed that 129 mice were older than C57BL/6Tac (p < 0.05), and both C57BL/6Tac and 129/SvEvTac were older than FVB/NJ (p < 0.001, MODLSD Bonferroni t-tests). Next, we compared the weights of hemi-brains used for the quantification of neurons. The comparison revealed no differences due to strain, genotype or gender sex (data not shown). Also, none of second and third order interactions between the factors was significant at α = 0.05. To avoid potential bias in the analyses of cell and neuronal counts between strains and genotypes due to existing subtle (not significant) differences in age ranges and brain weights, we investigated the relationships between age and brain weight, and total cell and neuronal cell counts. Since neither bivariate Pearson nor partial correlations, the latter controlling for the strain effects, revealed significant associations between the two independent variables and total cell or neuronal counts (data not shown), we did not include the age or brain weight as covariates in the analyses. Also, since the representations of males and females in some strain and genotype cohorts was small (2–3 mice per cell in most cases) and the preliminary data screen analyses revealed no significant effects of sex or interactions involving sex, we pooled that data across gender. Overall analysis of total cell and neuronal counts revealed a significant genotype effect (F(1,36) = 20.6, *p* < 0.001, η^2^ = 0.36, RMANOVA), cell type count (F(1, 36) = 2330.7, *p* < 0.001, η^2^ = 0.99), and cell type count by genotype interaction (F(1, 36) = 12.5, *p* = 0.001, η^2^ = 0.26). None of the other factors or interactions were significant at α = 0.05. Post hoc analysis revealed no significant strain effects or strain by cell type interaction for non-transgenic and TgTau^P301L^ mice (data not shown), indicating that the significant genotype effect was due to the differences in the cell number between nTg and TgTau^P301L^ mice, and not being affected by the strain of the line or interactions involving strain (Fig. 3).

Analysis of total cell counts revealed no significant effects of strain, transgenotype or the interaction between the factors (data not shown). However, there was a significant transgenotype effect (F (1, 36) = 44.9, *p* < 0.001, η^2^ = 0.56), with TgTau^P301L^ mice having overall fewer neurons than their non-Tg littermates (85.4 ± 2.4 versus 108.3 ± 2.4 per gram of brain wet weight, respectively). The average number of neurons per gram of brain weight, detected by means of counting NeuN positive nuclei, is presented in Fig. 3. The comparison between the genotypes within each genetic background revealed a consistent finding that TgTau^P301L^ mice had significantly lower number of neurons (t(12) = 2.3, *p* < 0.05, (t12) = 4.6, *p* = 0.001, and t(9) = 6.3, *p* < 0.001 for C57BL/6Tac, 129/SvEvTac and FVB/NJ backgrounds, respectively (degrees of freedom for FVB mice were adjusted due to inequality of variances)). Thus, on average, the three genetic backgrounds did not affect the degree of neuronal loss manifested by aged TgTau^P301L^ mice.

### Heterogeneity in tau pathologies

**Fig. 4:**
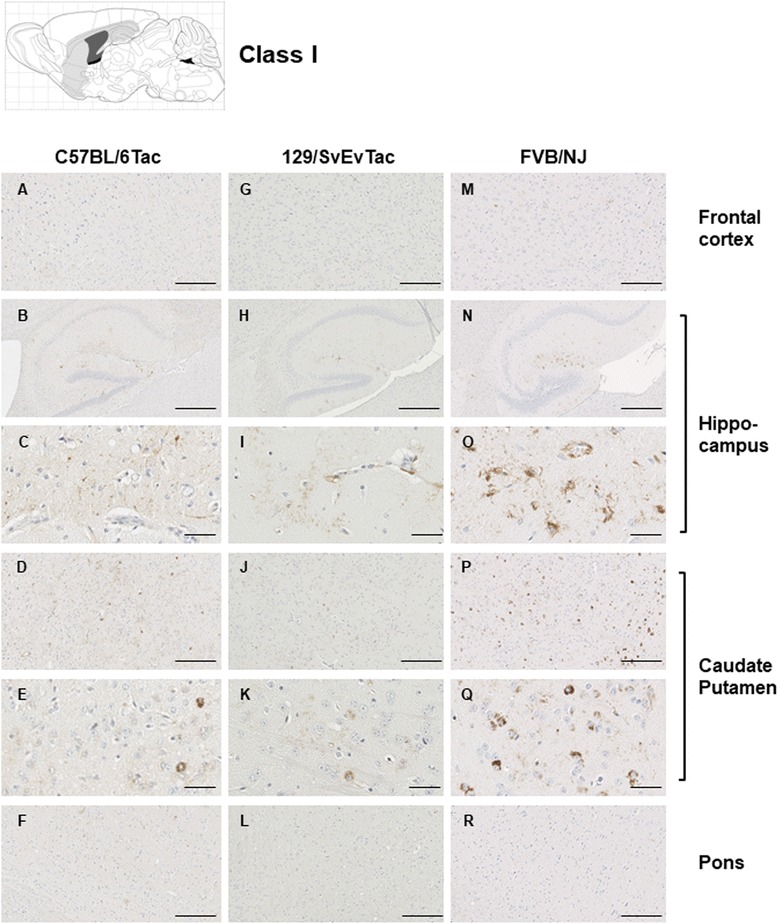
Class I Tau pathology. Immunostaining with the phosphorylation-dependent anti-Tau antibody AT8 in the brain of TgTau mice illustrating the different patterns of deposition. Three different genetic backgrounds are represented C57BL/6 J (panels **a** to **f**); 129/SvEvTac (panels **g** to **l**) and FVB/NJ (panels **m** to **r**). Individual animals of each inbred strain presented here (and also in Figs.5-9) with the different antibody analyses shown in Figs. S2-S17 representing probing of closely adjacent sections from the same animals as Figs. 4-9. Several structures characteristic of the pattern of deposition are shown: while the frontal cortex is negative (panels **a**, **g**, **m**), the hippocampus (“Hpc”, panels **b**, **c**, **h**, **i**, **n**, **o**) exhibits GFT’s and occasional NFT’s, the caudate putamen (**d**, **e**, **j**, **k**, **p**, **q**) exhibits NFT’s while the Pons (panels **f**, **l**, **r**) is negative for immunostaining. Scale bar = 500 μm for panels **b**, **h**, and **n**. Scale bar = 250 μm for panels **a**, **d**, **f**, **g**, **j**, **l**, **m**, **p**, **r**. Scale bar = 50 μm for panels **c**, **e**, **i**, **k**, **o** and **q**

**Fig. 5:**
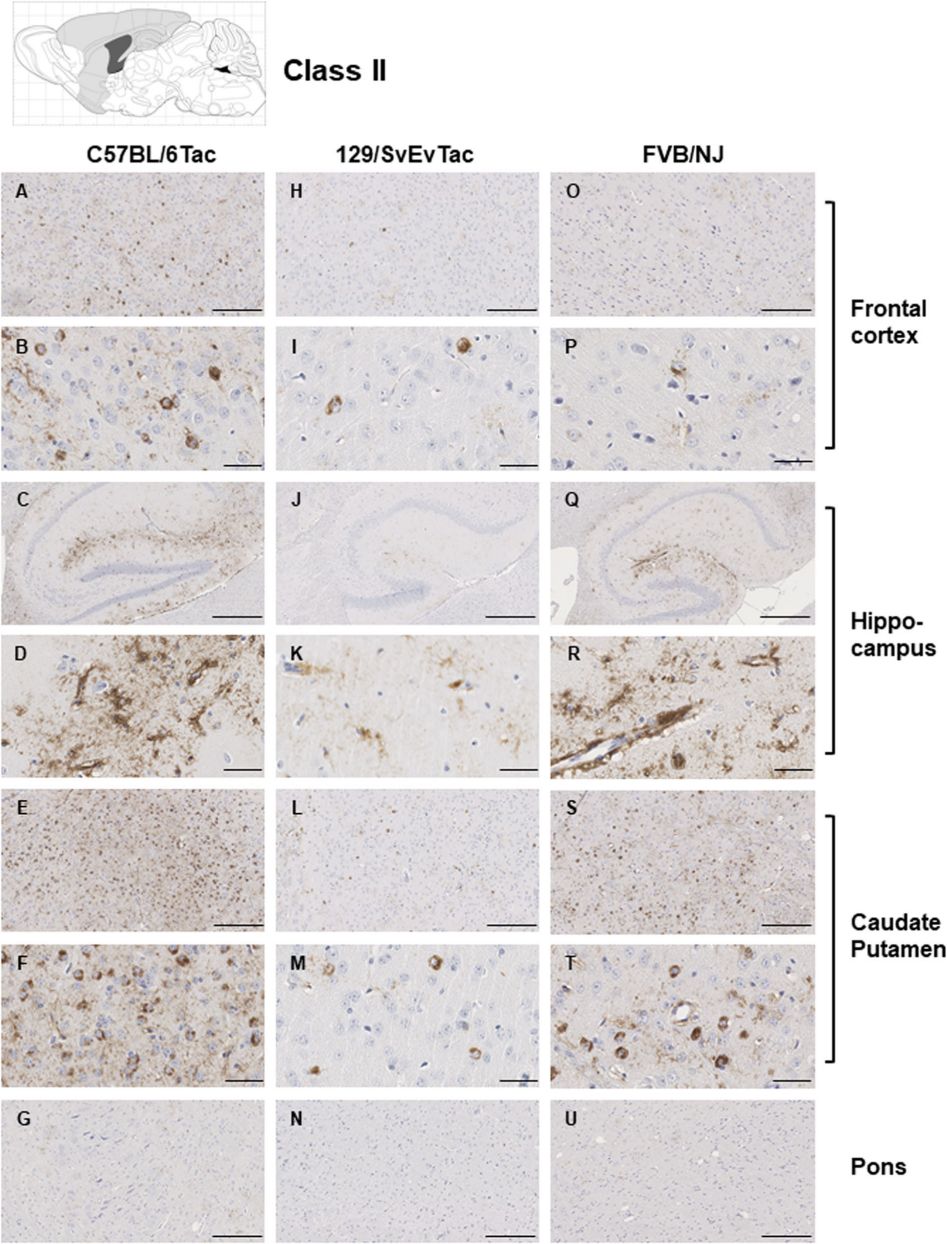
Class II Tau pathology. Three different genetic backgrounds have been studied with AT8 antibody as per Fig. 4. In distinction to class I there is staining in the cortex corresponding to NFT’s and GFT’s (**a**, **h**, **o**, **b**, **i**, **p**) but like class I has staining in hippocampus (panels **c**, **j**, **q**, **d**, **k** and **r**) and caudate putamen (panels **e**, **l**, **s**, **f**, **m**, **t**), with the pons being spared (**g**, **n**, **u**). Scale bar = 500 μm for panels **c**, **j** and **q**. Scale bar = 250 μm for panels **a**, **e**, **g**, **h**, **l**, **n**, **o**, **s** and **u**. Scale bar = 50 μm for panels **b**, **d**, **f**, **i**, **k**, **m**, **p**, **r** and **t**

**Fig. 6:**
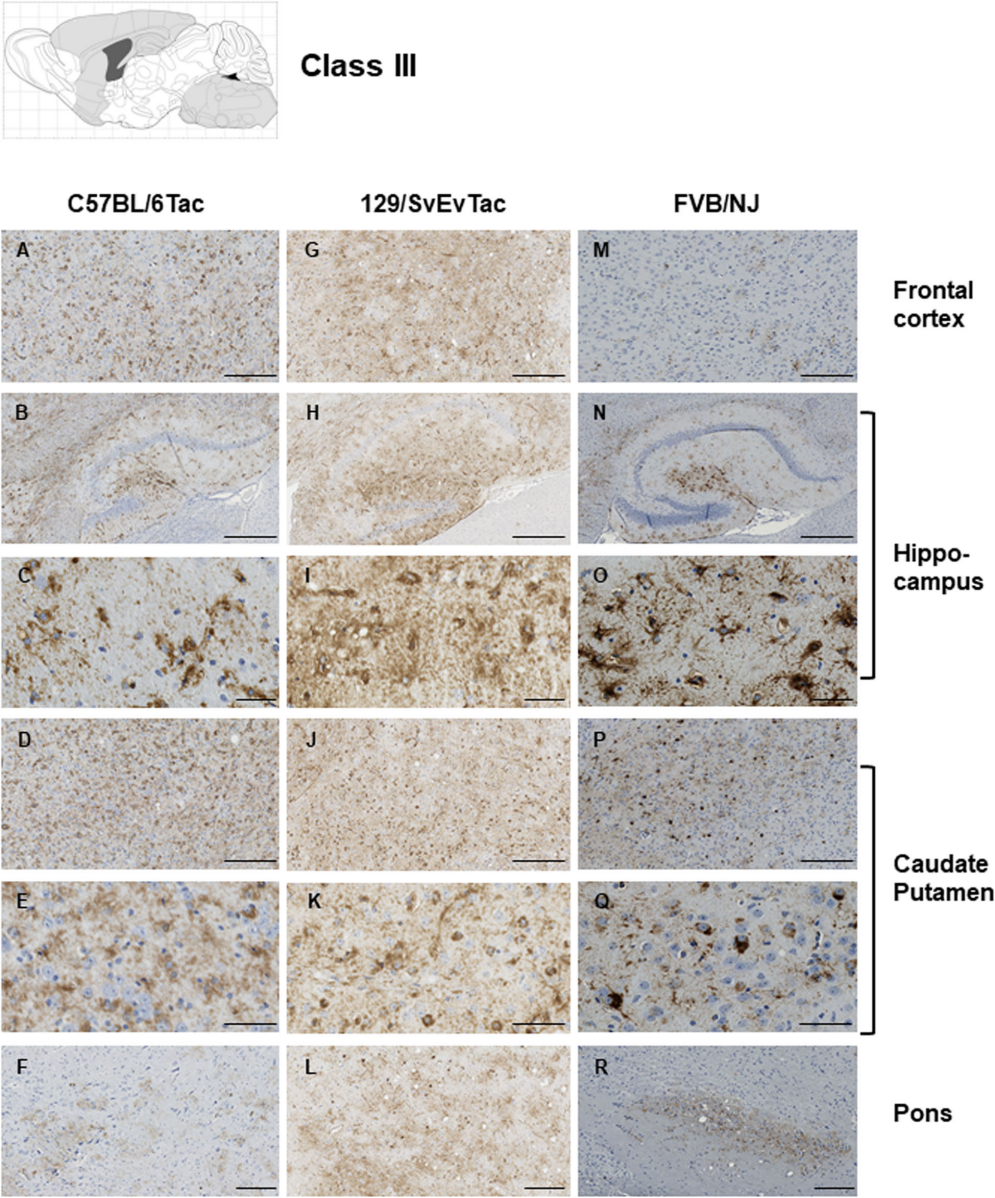
Class III Tau pathology. Different genetic backgrounds and anatomical structures have been studied with AT8 antibody as per Fig. 4. The distinction from class I and II is the involvement of the pons (**f**, **l**, **r**). Scale bar = 500 μm for panels **b**, **h**, and **n**. Scale bar = 250 μm for panels **a**, **d**, **f**, **g**, **j**, **l**, **m**, **p**, and **r**. Scale bar = 50 μm for panels **c**, **e**, **i**, **k**, **o**, and **q**

**Fig. 7:**
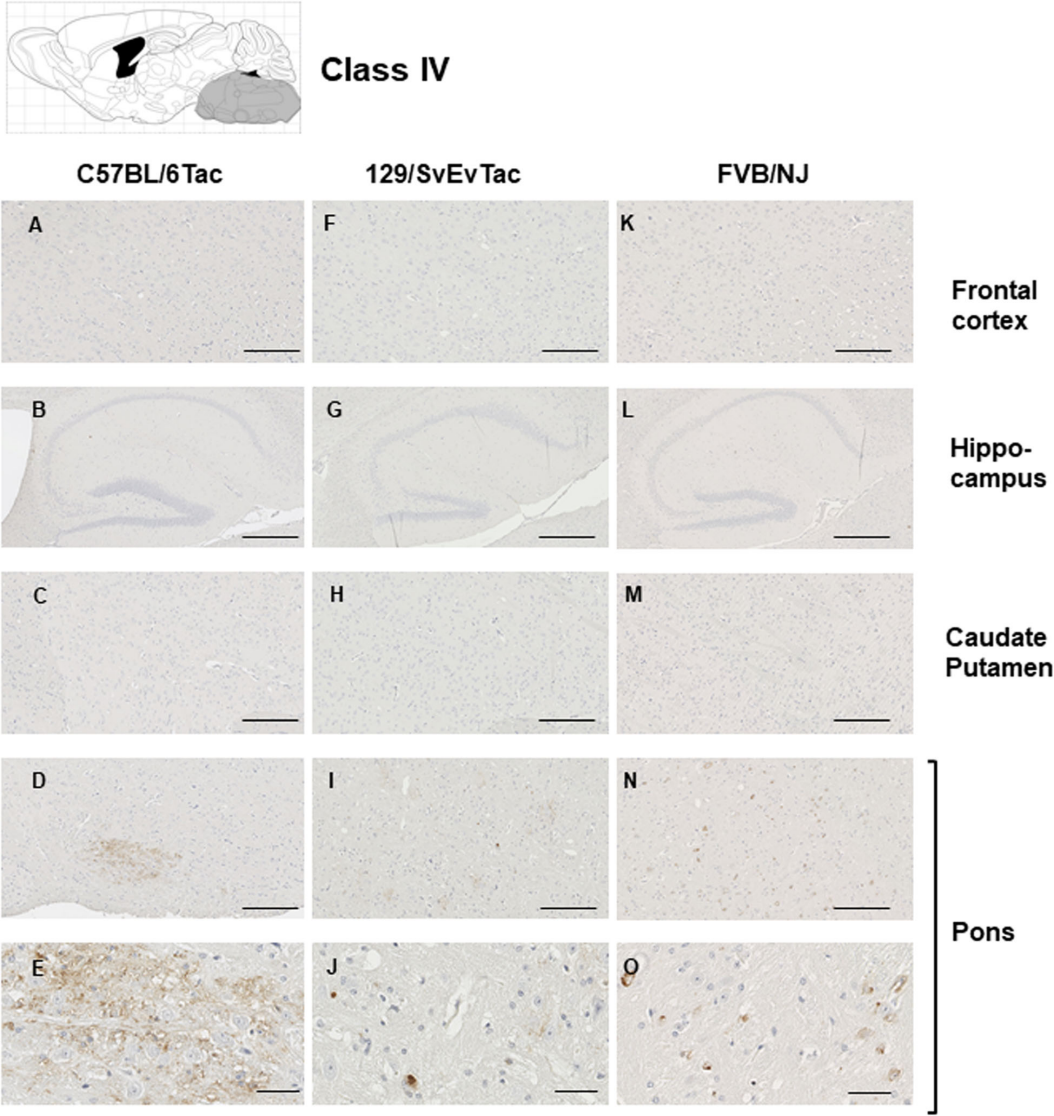
Class IV Tau pathology. Different genetic backgrounds and anatomical structures have been studied with AT8 antibody as per Fig. 4. In contrast to classes I-III, immunostaining is restricted to the pons (**d**, **e**, **i**, **j**, **n**, **o**). Scale bar = 500 μm for panels **b**, **g**, and **l**. Scale bar = 250 μm for panels **a**, **c**, **d**, **f**, **h**, **i**, **k**, **m**, and **n**. Scale bar = 50 μm for panels **e**, **j**, and **o**. Cells presented in panel **j** exhibit vacuoles

**Fig. 8:**
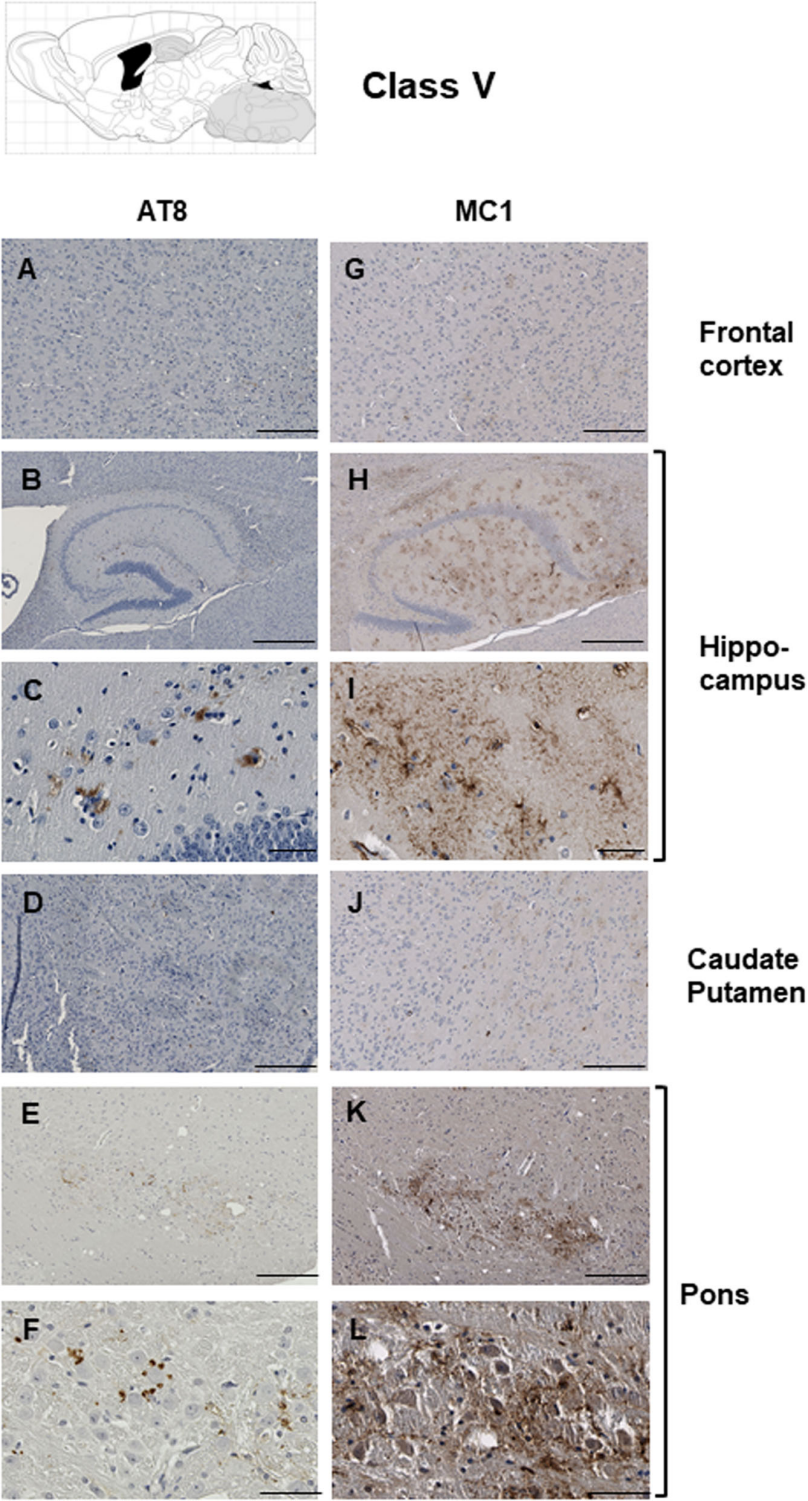
Class V Tau pathology. Class V comprised the rarest class of pathology. Three out of four occurrences were in the 129/SvEvTac background and a single mouse from this background is represented with AT8 or MC1 staining. In contrast to class IV, immunostaining encompasses the hippocampus (**b**, **c**, **h**, **i**). Scale bar = 500 μm for panels **b**, and **h**. Scale bar = 250 μm for panels **a**, **d**, **e**, **g**, **j**, and **k**. Scale bar = 50 μm for panels **c**, **f**, **i**, and **l**.

We next used histological analysis to see if the performance of the three inbred Tg derivative lines diverged as they aged. For this purpose, we used the AT8 anti-Tau antibody, a reagent highlighted in a consensus meeting for the diagnosis of FTD [34] and also used in the characterization of Tau strains in inoculation experiments [35]). Regarding Tg mice sampled in the range of 450–758 days of age, heterogeneity was manifest in several effects. From a sample of 113 AT8-positive aged TgTau^P301L^ mice, we encountered heterogeneities in deposition (Table 1). Of these, 84 brains were provisionally classified as belonging to one of five different categories, as scored by independent observers (Figs. 4, 5, 6, 7, 8). The five categories were present in each genetic background, with the exception of Class V, which lacked an example from FVB/NJ mice. To assess if the predominant categories (I-III) might represent different steps in a synchronous process, we examined their average ages of onset but here no pair-wise comparisons for sample groups >5 within the same inbred strain type reached significance (Table 1). Pooled across genetic backgrounds categories I-III were represented by both genders in a 1:15 ratio (32 females, 37 males).

**Table 1:** Pathology classes in TgTau^P301L^ mice analyzed by genetic background

^a^Genetic background (n ♀, ♂)	^b^Pathology Class average age in days ± SD (n♀, ♂)				
	I	II	III	IV	V
C57BL/6Tac (16,12)	^c^625 ± 90 (1,6)	^c^633 ± 60 (7,2)	^c^643 ± 77 (6,3)	472, 567 (1,1)	593 (1,0)
129/SvEvTac (11,18)	504, 662 (0,2)	^d^666 ± 60 (5,7)	^d^647 ± 61 (3,4)	541 ± 56 (3,2)	607 ± 73 (0,3)
FVB/NJ (12,15)	^e^583 ± 61 (2,8)	^e^597 ± 59 (3,3)	^e^615 ± 53 (5,2)	602 ± 102 (2, 2)	N/A (0)

Pathology classes were based upon the location of AT8 antibody immunostaining

^a^C57BL/6Tac age-range = 451–758 days; 129/SvEvTac age-range = 466–733 days; FVB/NJ age-range = 451–678 days. Aged animals lacking pathology are not included in this Table but are described in the main text

^b^All pair-wise comparisons of mean age for classes I-IV with data pooled from three genetic backgrounds were not significant (n.s.)

^c,d,e^All pair-wise comparisons of mean age within a genetic background n.s

Tg mice not present in the table include one C57BL/6Tac animal showing both class II and IV pathologies, one 129/SvEvTac animal showing both class III and IV pathologies while a further 27 animals did not fit within this classification scheme (12 C57BL/6Tac, 8129/SvEvTac and 7 FVB/NJ; see also Fig. 11)

N/A, not applicable

**Fig. 9:**
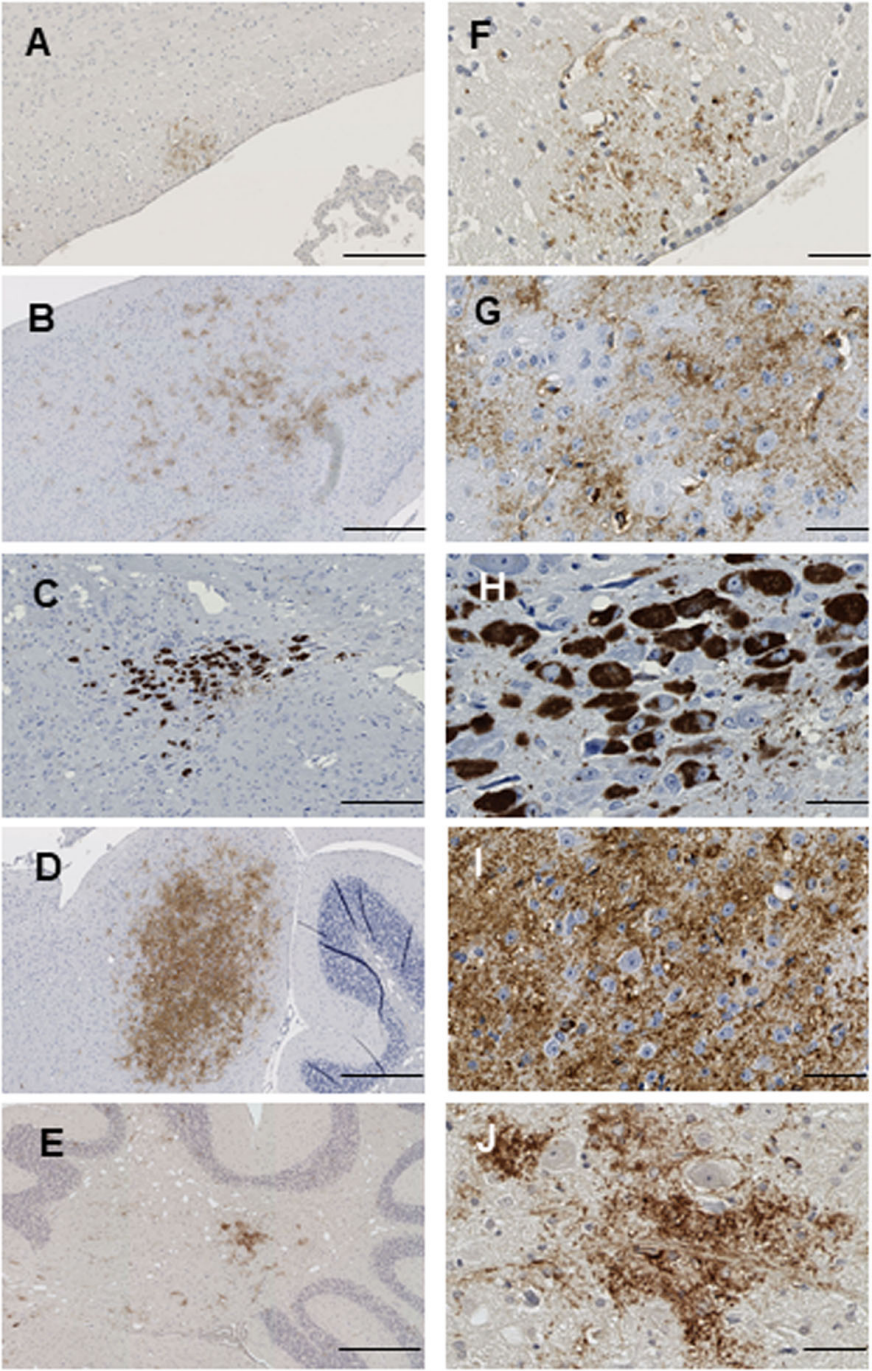
Focal staining in aged TgTau^P301L^ mice. Representative images of focal immunostaining of sagittal brain sections analysed with AT8 antibody. Corpus callosum (panels **a**, **f**), retrosplenial areas (panels **b**, **g**), Locus coeruleus (panels **c**, **h**), inferior colliculus (panels **d**, **i**) and cerebellar fiber tracts (panels **e**, **j**) are presented. Scale bar = 250 μm for panels **a**, **b**, **c**, **d**, and **e**. Scale bar = 50 μm for panels **f**, **g**, **h**, **i** and **j**

The features that formed the basis of this classification scheme are detailed in the following manner. ***Class I*** has striatal staining that includes mature NFT’s in the caudate and GFT’s in the hippocampal formation (Fig. 4). Cortical staining is minimal. ***Class II***: this is the most common class, representing close to half of the animals examined, having predominance of pathology in the frontal cortex but with staining in this area being augmented in other animals with striatal and hippocampal staining (as per Class I) and eventually encompassing all ventral brain areas (Fig. 5). This pattern of deposition resembles mice in our first description of the TgTau^P301L(T)^ line [12]. ***Class III***: this manifestation is defined by immunoreactivity in the pons and/or medulla occurring in addition to Class II reactivity (Fig. 6). ***Class IV*** represents a caudal pattern of deposition with rostral areas spared (Fig. 7) while in ***Class V*** animals this pattern is augmented by numerous GFTs in the molecular layer of the hippocampus (Fig. 8). Amongst mouse brains which did not fit within these classes, several exhibited strikingly focal patterns of deposition with the rest of the brain completely spared; five such patterns of focal deposition in corpus callosum, the retrosplenial area, the locus coeruleus, the inferior colliculus and the cerebellar white matter tract are presented in Fig. 9. Beyond AT8 antibody, we used conformation, phospho, and sequence dependent antibodies (MC1, PHF1, CP27 and RZ3) to re-examine brain sections from the five canonical classes (Additional file 3: Figures S2-S5, Additional file 4: Figures S6-S9, Additional file 5: Figures S10-S13, Additional fie 6: Figures S14-S17, Additional file 7: Figure S18). These analyses yielded generally similar results, albeit with a tendency for MC1 to yield superior signals in brain stem pathologies.

### Variable tau pathology versus transgene expression effects

**Fig. 10:**
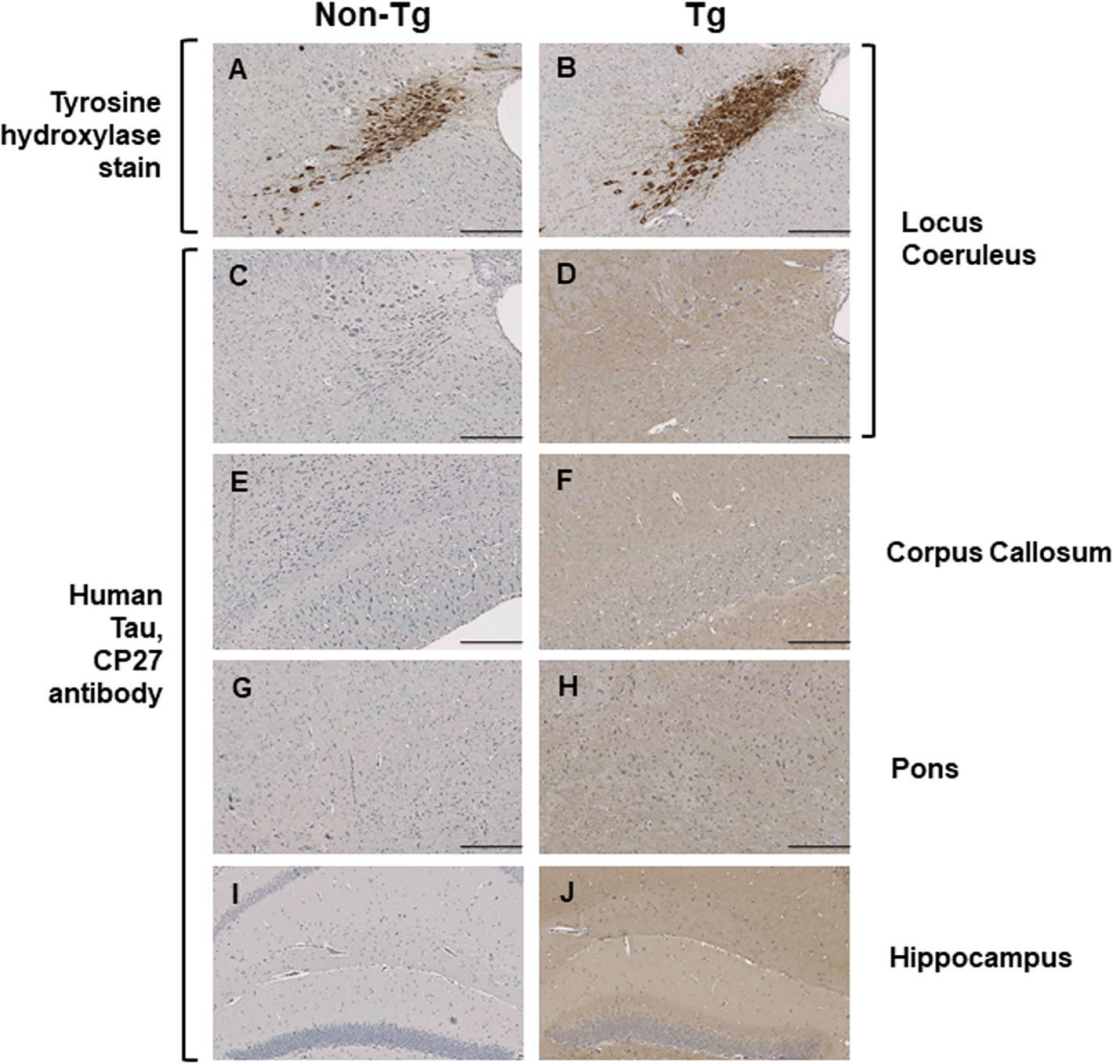
Transgene-encoded human Tau is not expressed in a focal pattern in areas subject to focal staining in aged mice. Neuroanatomical areas prone to focal deposition in aged Tg mice were assessed before the onset of AT8-positive immunostaining. (**a**, **c**, **e**, **g**, **i**) non-Tg and (**b**, **d**, **f**, **h**, **j**) TgTau^P301L^ mice (C57BL6/Tac background, ages 355 and 309 d, respectively) stained for tyrosine hydroxylase antibody in the locus coeruleus. The remaining panels indicate sections from different brain areas from non-Tg mice and Tg mice stained with using CP27 antibody, with Tg mice not exhibiting focal patterns of staining. **c**, **d**: locus coeruleus; **e**, **f**: corpus callosum; **g**, **h**: Pons; **i**, **j**: Hippocampus. All scale bars = 250 μm

**Fig. 11:**
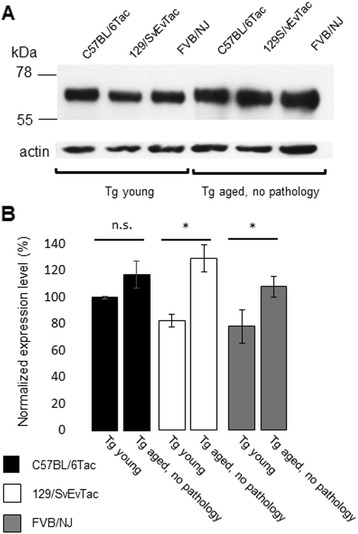
Presence of Tau protein in aged Tg animals with negative pathology. **a** Matching hemibrains of aged animals found lacking AT8 immunostaining (lanes 4–6, ages 723, 608, and 604 d respectively) were processed for western blot analysis and probed with antibody alongside young Tg mice (lanes 1–3, 66, 84 and 58d, respectively). Actin re-probe (lower panel) indicates similar sample loadings. **b** represents densitometric analyses of the blot data expressed normalized to actin and adjusted to C57BL/6Tac (“100%”). Aged pathology-negative Tg mice had more, rather than less, Tau than their young counterparts from the same inbred strain background, this reaching significance for the 129/SvEvTac and FVB/NJ backgrounds

Given the aforementioned heterogeneities in pathology, we performed further analyses to assess potential relationships to hypothetical variations in transgene expression. While dispersed patterns of transgene expression are visible at low magnification (Fig. 1d), we examined areas that defined pathology classes or were prone to focal pathology (Figs. 4, 5, 6, 7, 8, 9), to see if they were prone to corresponding hotspots of transgene expression. Using human-specific CP27 antibody to assess transgene expression in young animals, this type of relationship did not prove to be the case (Fig. 10, a-j), with dispersed immunostaining visible in these neuroanatomical areas. Secondly, we noted that Tau pathology was incompletely penetrant in aged animals; a lack of AT8-positive Tau pathology was apparent in about one quarter of all TgTau^P301L^ mice examined (32/145, range 451 to 758 days). Pathology-negative animals were present in each of the three genetic backgrounds as follows: 13/41 C57BL/6Tac Tg mice, average age ± SD = 614 ± 91 days; 7/36 129SvEv/Tac Tg mice average age 636 ± 33 days and 12/39 FVB/NJ Tg mice, average age 555 ± 58 days. However, this lack of pathology was not due to lack of transgene expression, as assessed by western blot analysis of the other brain hemisphere of animals assessed for AT8 immunostaining (Fig. 11); indeed, the aged Tg mice with no discernible pathology had immunoblot signals greater than their young counterparts. Both of these analyses argue against a crucial rate-limiting effect of transgene expression.

### Biochemical analyses of tau species in different pathology classes

**Fig. 12:**
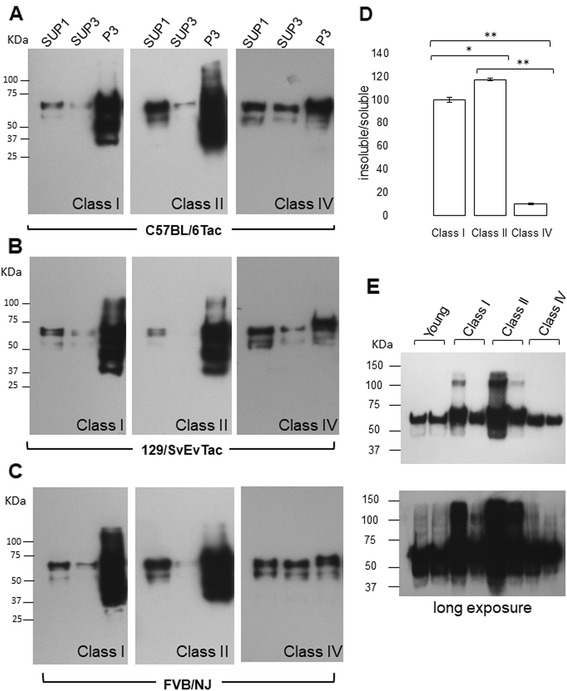
Insoluble Tau species in the brains of aged TgTau^P301L^ mice. Fractionated brains comprising supernatant pellet 1 (SUP1), supernatant pellet 3 (SUP3), and pellet 3 (P3) of aged TgTau^(P301L)^ mice were analyzed by western blot analysis. One example of Class I, II and IV is shown for each genetic background. **a** C57BL/6Tac mice at ages 587, 732, and 530 days left to right (**b**), 129/SvEvTac at ages 662, 592, and 466 days left to right, and (**c**) FVB/NJ mice at ages 646, 658, and 639 days left to right. For all samples, 10 μg of total protein was loaded on the gel. Antibody: CP13 (1/500; phosphoserine 202). SUP = Supernatant, and P = pellet fractions. **d** P3/S3 ratios of animals presented in A-C. Ratios were pooled across strain backgrounds. Class I vs. II *p* = 0.020; I vs IV, *p* = 0.08; II vs, IV, *p* = 0.003. **e** S1P fraction of animals with classes I, II and IV pathology to show the presence of oligomeric species in the soluble extract, antibody: CP27 (1/500)

Fractionation procedure presented in Fig. 2 were used to assess alterations in Tau species in Tg mice of each of the three inbred backgrounds in their second year of life (Fig. 12 **,** Additional file 8 **:** Figure S19**;** range 466-732d), examining Class I, Class II and Class IV animals. These analyses with AT8 antibody confirmed the presence of phosphorylated Tau, especially 64–68 kDa species, in all brain fractions (SUP1, SUP3, and P3). There was a continuing trend in all three genetic backgrounds for insoluble Tau to accumulate in pellet 3 (P3) fractions of aged Tg mice versus mice at 240 days (Fig. 12 a-c, Fig. 2). Comparing amongst these aged animals, the yield of insoluble/soluble Tau (P3:SUP3) increased between Class I and II, with Class IV being significantly lower than either (*p* < 0.01); Fig. 12d). A further distinction from brain lysates of 240 days old mice was the presence of both lower and higher molecular weight bands in mice with class I and class II pathology; this was manifest in all three backgrounds (Fig. 12a-c). The lower molecular weight species may be due to fragmentation (as observed in FTDP-17, as well as AD patients’ brains [36–38]) while the higher molecular weight species with mobility above full-length transgene encoded Tau in Class I and Class II P3 samples were inferred to represent SDS-resistant aggregates. To investigate the possibility of oligomeric species further, the supernatant of P1 samples (SUP1) was subject to brief ultracentrifugation and the concentrated samples analyzed on denaturing gels (Fig. 12e). For three out of four Class I and Class II samples, signals were seen at a mobility slower than a 100 kDa M_r_ marker. Conversely, such signals were absent from equivalent Class IV samples, even upon extended autoradiographic exposure. Probing of P3 fractions with CP13 total tau and PHF phospho-Tau antibodies revealed overlapping signatures for Classes I and II mice, albeit with different relative intensities for putative fragmented species (in the range 30–50 kDa) for CP13 vs. PHF1 analyses (Additional file 8: Figure S19). Class IV animals presented with weaker, less complex signals that in two cases included prominent species at 50 and 25 kDa.

**Fig. 13:**
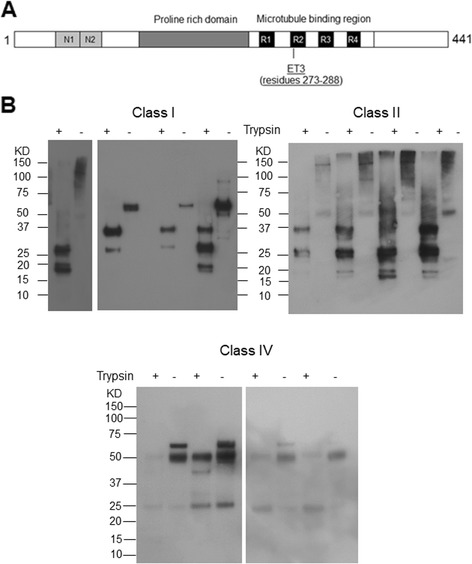
Trypsin-resistance of sarkosyl-insoluble Tau fractions. **a** A schematic of antibody epitope is presented. **b** 10 μg of P3 fractions from animals of classes I, II and 15 μg of P3 for animals of IV were subjected to trypsin digestion (1/100 for enzyme/protein ratio) and analyzed by western blotting. The banding patterns in samples are represented before and after trypsin-digestion. The samples are organized by age in an increasing order. Animals from class I, left to right C57BL/6 J, FVB/NJ, two C57BL/6 J and FVB/NJ. Animals from class II, left to right C57BL/6 J, two 129/SvEvTac, FVB/NJ and C57BL/6 J and 129SvEv/Tac and two FVB/NJ and C57BL/6 J animals from class IV. The exposure time for different paired samples electrophoresed and transferred from the same gel was adjusted to obtain similar signal intensities for the predominant immunoreactive species. ET3 anti-Tau (4R specific, residues 273–288) was used to detect Tau fragments at 1/250 dilution

We performed trypsin digestions on P3 fractions of Classes I, II and IV mice (Fig. 13, *N* = 4); these samples were probed with an antibody which has an epitope lying within a previously defined trypsin resistant core, ET3 [23]. Strikingly, these digests yielded three types of signature: i) in Class I, a prominent band at ~37Kda and a weaker band at 27 kDa (N = 4), ii) in Class II, a double band (“doublet”) at ~26 and 28 KDa and another doublet at 20 and 18 kDa (*N* = 4), and iii) in Class IV, a stronger band at 53 kDa and a weaker band at 25 kDa in (*N* = 4). One mouse classified as Class II had an intense signal at 37 kDa suggestive of a blended Class I + Class II signature, as did one Class I mouse. One Class I mouse had a signature lacking a 37 kDa band and more similar to Class II (Fig. 13b). The finding of seemingly related Class I and Class II signatures and yet distinct Class IV signatures was reiterated in analyses of undigested P3 samples probed with CP13 and PHF1 antibodies (Additional file 9: Figure S20). Also, in these analyses either with or without trypsin treatment, Class IV animals often yielded weaker signals than those obtained for Classes I and II.

### Electron microscopic analysis of tau filaments

**Fig. 14:**
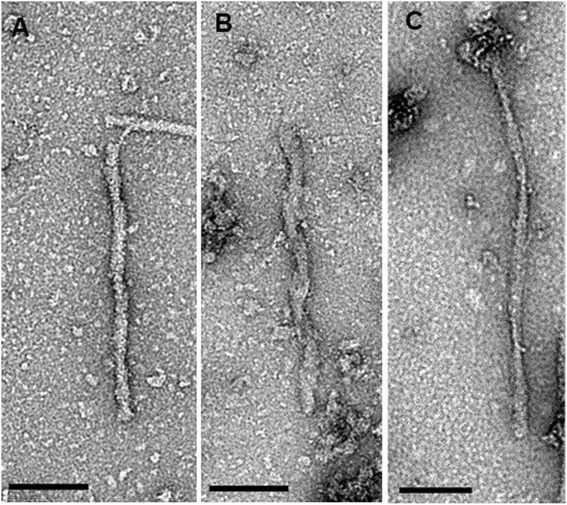
Negative stain electron microscopy of insoluble Tau fractions. The morphology of individual Tau filaments was readily discernible and three separate filament types were observed: straight filaments (**a**), coiled filaments (**b**), and twisted ribbon-like filaments (**c**). Scale bars = 100 nm

We next analyzed the ultrastructure of individual Tau filaments in the P3 fractions (Fig. 14). Negatively stained Tau filaments were readily apparent in most of the P3 fractions available for electron microscopy (Table 2). The morphology of individual Tau filaments fell into three recognizable types: straight filaments (Fig. 14 a), coiled filaments (Fig. 14 b), and twisted ribbon-like filaments (Fig. 14 c). Only a few filaments could not be assigned to one of these three types due to overlapping particles or poor staining (not shown). In all samples, the straight filaments were the dominant type, coiled filaments were most noted amongst Class I samples, while twisted ribbon-like filaments were rare in all samples (Table 2). The number of observed filaments was disproportionately lower in all Class IV samples, suggesting that only a lesser fraction of the Tau protein had fibrillized as compared to Classes I and II.

**Table 2:** Categorization of individual Tau filaments according to pathology classification and genetic background

Mouse lines	Pathology Class I				Pathology Class II				Pathology Class IV			
		Filament Types				Filament Types				Filament Types		
	N	Straight Filament	Coiled Filament	Twisted Ribbon-like Filament	N	Straight Filament	Coiled Filament	Twisted Ribbon-like Filament	N	Straight Filament	Coiled Filament	Twisted Ribbon-like Filament
C57BL/6	2	39	35	1	2	189	0	0	2	6	0	0
		52%	47%	1%		100%	0%	0%		100%	0%	0%
129SvEv	1	22	0	1	1	55	0	0	3	23	0	0
		96%	0%	4%		100%	0%	0%		100%	0%	0%
FVB/NJ	2	21	0	0	3	121	1	0	3	14	0	0
		100%	0%	0%		99%	1%	0%		100%	0%	0%

Negative stain electron microscopy was used to distinguish the different morphologies of isolated Tau filaments (Fig. 14). Filaments numbers were totaled for animals of the same Class and inbred strain type. N, number of animals analyzed

### Seeding activity in brains from aged Tau^P301L^ mice

**Fig. 15:**
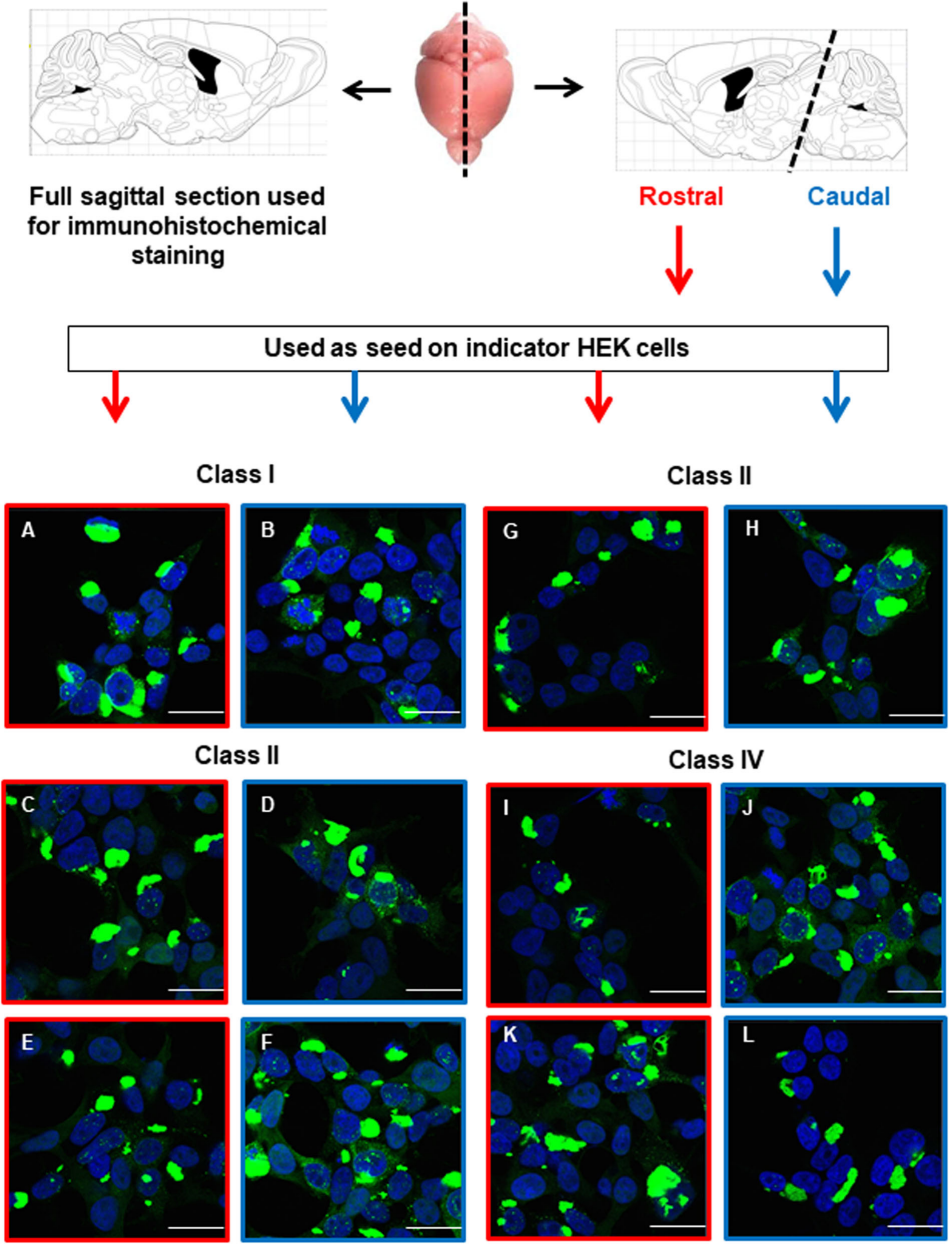
Assessment of Tau strains in a cell-based seeding assay. Upper panel: Mouse brains have been cut saggitaly with the right hemispheres fixed in formalin for further processing and embedding in paraffin and for use in immunohistochemistry. Left hemispheres were cut transversally according to the diagram (dashed line). Each rostral or caudal portion was then homogenized and used as a seed on cell cultures as described. Lower panels represent the fluorescent micrographs obtained from seeding assays with rostral (red border) or caudally-derived (blue border) derived homogenates. When transduced into Clone 1 cells, which express Tau RD-YFP but lack aggregates, all homogenates seed morphologically indistinguishable Tau inclusions, which feature tangles of filamentous Tau (panels **a**-**l**). These inclusions are morphologically distinct from those seeded by Clone 9 (nuclear speckles) and Clone 10 (ordered inclusion) lysates (see Additional file 10: Figure S21)

Lastly, we asked whether the different patterns of deposition in individual mice were due to structurally distinct self-propagating Tau amyloid conformers or “strains” (strains used here in the sense of prion effects). Previously, a monoclonal cell culture model was developed that could differentiate Tau strains based on inclusion morphology [27]. These recipient cells (Clone 1) stably express the Tau repeat domain (“RD”, amino acids 244–372 of the longest 441 amino acid 2 N,4R isoform) fused to YFP. At baseline, Tau RD exists as soluble monomer. However, upon addition of exogenous aggregates or seeds, cells rapidly convert to an accumulation of a fibrillar state, which propagates itself stably to daughter cells over months of culture. In this paper, two recombinant fibrillar Tau-derived strains (Clone 9 and Clone 10) were extensively characterized. Consistent with earlier results, addition of Clone 1 lysate did not seed aggregation (Additional file 3: Figure S3a**)**. However, Clone 9 and Clone 10 lysate seeded aggregation in the majority of cells and produced characteristic inclusion morphologies—Clone 9, nuclear speckles (Additional file 3: Figure S3b); Clone 10, an ordered juxtanuclear inclusion (Additional file 3: Figure S3c). Using rostral and caudal samples derived from a total of six mice classified as having Class I, Class II or Class IV pathology, we tested seeding capacity. All 12 TgTau^P301L^ mouse brain samples seeded inclusion formation (Fig. 15, a-l), with a similar signature featuring large deposits of Tau tangles with few nuclear deposits; these inclusions thus differed from those associated with Clone 9 and Clone 10. We conclude that a single Tau species predominates in these seeding assays, irrespective of the location and intensity of Tau deposition. In terms of strain identity, out of a previously defined panel of 18 possible Tau strains deriving from diverse source materials (recombinant Tau fibrils, cell lines, human brain material from different Tauopathies, mouse brain material), the signature morphology from TgTau^P301L^ mice most closely resembled a strain isolate called DS6 [35]. Interestingly DS6 derives from a homogenate of a P301S Tau Tg mouse brain [39]. We conclude that seeding activity does not necessarily correspond with areas of Tau immunostaining.

## Discussion

### Phenotypic variation and classes in P301L Tauopathy

Our earlier studies defined a degree of diversity in cognitive and pathology phenotypes in a P301L mouse model [12] and we have now sought to understand these phenomena within the framework of an extended study. New derivative sublines of TgTau^P301L^ mice were created in three distinct inbred backgrounds, and, while uniform responses were recorded in some criteria, neuropathological diversity was certainly not extinguished. Rather, the larger cohorts of animals produced for study allowed us to perceive both recurrent patterns (classes of pathology) or variations (animals either falling outside of the classification scheme with focal pathologies or completely lacking pathology) that we had not previously appreciated. Collectively, these data pose a number of overlapping questions that include a) the nature of operational parameters that distinguish this study from other studies, b) the mechanistic origin(s) of histopathological heterogeneity that includes stereotypic and variant, focal events, c) the possible role of “spreading effects”, d) the possible role of Tau “strains” and e) the possible relationships to clinical variations amongst patients with the same *MAPT* mutations.

### Signatures of uniform expression and protein misfolding in TgTau^P301L^ mice

Operationally, the paradigm described here is markedly different from using specialized promoters or stereotaxic injections of misfolded Tau to produce local effects [40–43]. But how might focal effects originate and could they relate to the transgene system? Besides special chemical attributes of the 2 N, 4R Tau isoform of human Tau, several factors may allow the accretion of diverse, focal pathology. First, the ability to accumulate Tau in neurons or astrocytes or oligodendrocytes [12], perhaps reflecting a low-level of “leaky” non-neuronal expression from the hamster PrP gene promoter or the trafficking of nascent, misformed Tau from neurons to astrocytes [44]. Second, the level of transgene expression (1.7X endogenous) may be fortuitous, allowing us to perceive focal events in a slow pathogenic cascade with most alterations occurring in the last 40% of the animals’ natural lifespan (using the aforementioned figure that 750 days of age represents 95% or greater of natural lifespan for inbred mouse strains that are not prone to neoplasias). These events cannot be scored in models with 1× expression such as P301L knock-in mice that have a latent underrepresentation of neuronal mitochondria but lack CNS pathology [45, 46], or might be difficult to discern in TgTau mice with overexpression, e.g. up to 13× in widely-used rTg4510 mice with loss of 23% of CA1 hippocampal neurons evident within the first third of a lifespan [47]. In the studies here, the TgTau^P301L^ transgene array is co-expressed with endogenous mouse *Mapt* and therefore approximates to the human situation with dominant *MAPT* mutations co-expressed with a WT allele. Misfolded human Tau species may be interacting with endogenous mouse Tau protein [48] but this would be a systematic effect and other studies have indicated that use of a *Mapt* ^0/0^ background blunts rather than exacerbates Tau toxicity [49]. Although epigenetic effects can affect neurobiological endpoints, mechanistically these derive from DNA and histone methylation modulating transcription [50] and the Tau transgene insertion site would need to be posited as a target for modification. In practice, the levels of Tau expression do not decrease with age in TgTau^P301L^ mice and do not seem to be a limiting variable for the development of Tau pathology, as shown by pathology-negative but transgene-expressing mice. Instead, broadly homogeneous expression in a rostral/caudal plane (Fig. 1d), as driven by the cos.Tet vector [51] is consistent with position-independent, pan-neuronal expression of transgene-encoded mRNA observed in other uses of this vector [29, 52, 53]; in this case it produces a comparatively uniform level of Tau substrate considered in a neuroanatomical sense (Figs. 1, 2) that may be conducive to scoring age-related events that cause focal deposition. Other studies failed to reveal hotspots of transgene expression (Fig. 10), again speaking against a rate-limiting role for expression of full-length 2 N, 4R Tau.

Foci can frequently initiate as astrocytic plaques in the molecular layers of the Hpc and the subiculum. In the pons, events can occur in the Raphe nuclei and superior olivary complex. In the striatum, foci occur as tangles and astrocytic plaques. Rarer events portrayed in Fig. 9 include the locus coeruleus and also the retrosplenial cortex (both known to capable of supporting the DS6 strain of misfolded Tau [35] - see below). Other animals fell outside of this classification scheme by having focal pathologies and yet other animals has no discernible Tau deposition. As the three inbred derivatives of Tg founder each approach genetic homogeneity and are different from each other (apart from the chromosome region flanking the insertion site and some non-syntenic blocks of DNA), there are strong indications that genetic effects do not underpin the pathologies that, instead, appear to define heterogeneous and possibly stochastic events. In this respect, our data parallel studies where FVB/129 versus C57BL/6NTac genetic backgrounds minimally altered the presentation of Tau pathology in rTg4510 Tau mice with a P301L mutation [54].

### Classes of pathology and tau spreading

When Tau attains abnormal forms, and becomes fragmented in older animals (as per Fig. 12 a-c), this may facilitate template-directed folding and neuroanatomical “spreading” in a prion-like manner [55–58] to produce a larger field of pathology. Hence, deposition in animals with higher burden of pathology could reflect the sum total of early initiating events plus subsequent events derived from spreading. Given the involvement of adjacent fields, Classes I, II and III could be interpreted to represent sequential invasion of neuroanatomic areas from an initial focus, but the scenario with specific classes synchronized to specific chronological ages is excluded as these classes have indistinguishable average onset within an inbred strain and between each other (Table 1). In this respect, our data differ from analyses of compound Tg mice comprised of PS19 Tg mice (1 N, 4R isoform) with a frontotemporal dementia mutation (P301S) [39] crossed with PDAPP Aβ-depositing mice [59]; the majority of these animals represented predictable progression between the ages of 4, 8 and 11 months from stages referred to as I/II, III/IV and V/VI [60]. Cortical deposition was present in all stages and some analogies to the Braak staging scheme for sporadic AD were also noted [60–62]. On the other hand, for TgTau^P301L^ mice, if initiating focal events were to start at different ages in different animals, then a predictable progression from Class I- > II- > III promoted by “spreading” becomes plausible. In this scenario, two animals assigned to different classes - I and III, for example - could nonetheless share the same chronological age. A kinship between events in Class I and Class II mice is also suggested by biochemical analyses of Tau species (Figs. 12, 13 **,** Additional file 9 **:** Figure S20, Table 2). With regards to seeded growth as a necessary step in neuroanatomical spread, it is notable that brain material taken from older animals has seeding capacity in a fluorescent assay (Fig. 15 and discussed further below**)**. The low level of transgene expression in TgTau^P301L^ mice must limit the substrate concentration for templated refolding and in this sense, may be the key mechanistic variable to dictate “slow motion” pathogenesis and hence the ability to capture focal pathologies before spreading.

### Pathological heterogeneity and tau strains

For protein-mediated effects, templated growth of heterogeneous misfolded protein conformers might cause heterogeneity in neurobiological endpoints, a concept pioneered for prion infections. Prion strains involve differently misfolded forms of the cellular prion protein [63, 64] and prior analyses have suggested the existence of Tau “strains” in human brain material [27, 57]. However, the inventory of covalent modifications to Tau is complex, including alternative splicing of N-terminal sequences and/or microtubule binding repeat exons, truncation, phosphorylation at up to 85 sites, O-glycosylation and acetylation [65]. This complexity in covalent structure thereby imposes constraints upon interpreting Tau strain phenomena as an exclusive basis for pathological heterogeneity. Here we used a transgene construct with no alternative exonic splicing and used seeding assays and biochemical profiling to begin to address this issue.

Although the cellular assay for seeding was positive from Tg mouse-derived material and can detect up to 18 distinct Tau signatures [27, 35], we obtained only one signature from animals with rostral or caudal Tau deposition (i.e. Classes I, II or IV; Fig. 15). This particular signature resembled the isolate designated DS6 derived from P301S mice [35, 39]. Of note, DS6 produces deposits in many brain regions after inoculation [35], an observation compatible with the DS6-like seeding activity within TgTau^P301L^ animals populating different brain regions. In our experiments seeding activity was unrelated to the presence of AT8 immunostaining and it is unclear whether dispersed oligomeric species [66, 67] contribute to the seeding activity because these species were not readily detected in Class IV animals (Fig. 12d). However, widespread seeding activity with little relationship to protein deposition assessed by light microscopy clearly parallels results using a templating assay for mice expressing a mutant form of PrP [68]. Seeding activity is known to occur in early Braak stages of Alzheimer’s Disease [69] and given the observation of seeding before immunopositivity in TgP301S mice [70] it is possible that seeding activity arises early in the life of TgTau^P301L^ mice.

What about other signatures that might relate to Tau strain phenomena? Using EM to examine P3 samples we observed a predominant morphology of straight filaments, irrespective of class type (Table 2), similar to other analyses [5]. But in some other respects Class IV animals did differ from Classes I and II. The P3 fractions had the fewest observable straight filaments per μg of protein, generally less net immunoblot signal, different immunoblot quality as assessed with three antibodies and different signature after trypsin digestion (Figs. 12, 13 **,** Additional file 8 **:** Figure S19; Table 2). Some of these qualitative different properties may derive from a fundamentally different (lowered) propensity of misfolded Tau in Class IV animals to aggregate. In sum, TgTau^P301L^ mice clearly harbor at least three and perhaps up to four variants of Tau. One variant is detected in the seeding assay (this is present in all classes and is also present in brain areas without immunostaining), while three more variants are detected by trypsin digestion. In the absence of divergent data from seeding assays or true-breeding of biochemical properties in serial transmissions experiments we cannot formally conclude that variants reflect the creation of new Tau strains; however, this is a straightforward explanation of the current data.

### Origins of variegated tau pathology in inbred mice

The quantitative and qualitative differences in Tau species between the pathology Classes are unequivocally striking and they do beg the question of origination. Genesis of Tau seeding activity may be amenable to study in the congenic models presented here but beyond this, the macroscopic assemblies detected with anti-Tau antibodies represent independent and perhaps more biologically impactful events. Weaker/stronger AT8 reactivity in TgTau^P301L(T)^ mice was associated with lesser/greater amounts of insoluble Tau and better/worse performance in the conditioned taste aversion (CTA) assay for memory function [12]. Although CTA assays were not undertaken for the current cohorts, the correlation between Tau aggregation/deposition, synaptic dysfunction and frank cognitive impairment is well established [62, 71–75]. Hence a more pertinent question is “how can heterogeneity occur in aged brains, given the genetic constraints, the absence of extraneous pathogens and the regulated housing conditions?”. While the performance of other mouse models with indolent Tau deposition remains to be assessed, the question about heterogeneity is underscored by the benchmark of human case material, where phenotypic variation exists in human FTDP-17 *MAPT* kindreds, and can be present even within families harboring the exact same mutation in codon 301 [1, 14, 15, 17, 76–78].

Decreases in quality control processes affecting protein maturation, turnover and clearance might facilitate the appearance of abnormal Tau species that can then go on to initiate a cascade of accumulation [79–81] but the associated molecular and cellular pathways may be generic in scope - for example being present in immortalized mammalian cells and conserved in yeast - and not tailored to different neuroanatomical regions. Also, the proteostatic machinery of cells in a given neuroanatomical region might be anticipated to be similar between animals of the same age and genetic background. We therefore posit that external insults to the aging CNS - especially those that could act with asymmetry with respect to neuroanatomy - are the missing links needed to understand variegated Tau pathology. In principle, these insults/parameters could include: mechanical aspects of the blood supply such as focal alterations in the blood-brain barrier or hyper-vascularization, blood constituents such as hormones/cytokines/nutrients/toxins, as well as blood-borne cells that can patrol the CNS, such as microglia. Xenobiotics, such as metabolites from microbiota and variations in the microbiota themselves might also warrant consideration [82–88]. Here, the involvement of caudal brain structures (Figs. 7, 8, 9, 10, Additional files 6, 7, 8) that include inputs from the enteric nervous system and hence the digestive tract, illustrate a subset of these possibilities. Overall, we deduce that the initiating parameters that determine presence/absence of focal pathologies lie beyond the information stored in the mouse nuclear genome and beyond the internal CNS connectivity that allows Tau spreading [57]. The congenic models described here with indolent changes in pathology constitute experimental platforms to define and push stochastic, rate-limiting steps in pathogenesis in the aging mammalian brain. Understanding stochastic events in TgTau^P301L^ mice may be useful in understanding sporadic events that feature in the common idiopathic forms of dementia.

## Conclusions

In our first description of a TgTauP301L transgenic line, onset of clinical disease differed between Tg colonies maintained at different laboratory sites. Additionally, some heterogeneity also existed within cohorts of age-matched Tg mice of the same colony with regards to levels of insoluble tau and memory function [12]. Given that phenotypic variation occurs in patients carrying the same P301L mutation [1, 14–17], we extrapolated that the TgTauP301L line might be manifesting a related biological effect. Here we considered two views to account for this phenotypic heterogeneity; the action of genetic modifier loci versus a role for stochastic cell biological events and/or environmental inputs. To explore these hypotheses a founder stock designated TgTauP301L(T) was used to make three inbred derivatives. Phenotypic variations were not eliminated by this maneuver and different classes of pathologic variation were observed; these were shared between (and independent of) the three inbred genetic backgrounds, suggesting the action of extrinsic disease modifiers. Moreover, distinct molecular signatures for three pathology classes following trypsin digestion of detergent-insoluble material strongly suggest the de novo formation of different Tau strains. Thus, heterogeneous factors affecting the genesis of abnormal Tau species may be amenable to discovery using the inbred models described here.

## Additional files

Additional file 1: Table S1. Single Nucleotide Polymorphism (SNP) profiling of congenic and incipient congenic lines. (DOCX 11 kb)

Additional file 2: Figure S1. Appearance of disease-associated symptoms in TgTau^P301L^ mice three genetic backgrounds. Time points when disease-associated symptoms were apparent in animals are presented alongside the performance of non-Tg littermates. Survival curves of non-Tg littermates of Tg animals are represented as well. The symptoms recorded for Tg animals are specific for them and none of the non-Tg littermates manifest such symptoms. Non-Tg animal live up to ca. 750 days, however a few animals succumb to natural deaths or have health problems (such as dermatitis or eye infection), requiring euthanasia to be performed. The number of cohort sizes for each group (n) are represented within each graph. All sample groups were terminated at 750 days. (TIFF 83 kb)

Additional file 3: Figures S2-S5. Class I mice. These figures represent the counterparts of Fig. 4. stained with MC1, CP27, RZ3 and PHF1 antibodies, respectively. (ZIP 2909 kb)

Additional file 4: Figures S6-S9. Class II mice. These figures represent the counterparts of Fig. 5. stained with MC1, CP27, RZ3 and PHF1 antibodies, respectively. (ZIP 3613 kb)

Additional file 5: Figures S10-S13. Class III mice. These figures represent the counterparts of Fig. 6. stained with MC1, CP27, RZ3 and PHF1 antibodies, respectively. (ZIP 3238 kb)

Additional file 6: Figures S14-S17. Class IV mice. These figures represent the counterparts of Fig. 7. stained with MC1, CP27, RZ3 and PHF1 antibodies, respectively. (ZIP 2280 kb)

Additional file 7: Figure S18. Class V mice. This figure represents a counterpart of Fig. 8 stained with CP27, RZ3 and PHF1 antibodies. (TIFF 690 kb)

Additional file 8: Figure S19. Pathology in aged Tg mice assessed for insoluble Tau species. These data represent the counterparts (other hemi-brains) of the animals assessed for insoluble Tau species in Fig. 12; pathology class and genetic background are annotated. Scale bars for low power views = 2.5 mm, high power views = 50 μm. (TIFF 741 kb)

Additional file 9: Figure S20. Undigested P3 fraction assessed with CP13 and PHF1 antibodies. A schematic of antibody epitopes is presented. Blot represents P3 fraction from 3 animals of classes I, II and IV. Class I mice at ages 587, 662, and 646 days left to right, class II animals at ages 735, 592, and 658 days left to right, and class IV mice at ages 530, 466, and 639 days left to right. For both blots, 5 μg of total protein was loaded on the gel. Antibody: CP13 (1/500) and PHF1 (1/500). (TIFF 199 kb)

Additional file 10: Figure S21. Clones used for fluorescence microscopy assays. Supplement (A-C). Clone 1 (negative control) lysate never seeds inclusions, whereas Clone 9 and Clone 10 seed the formation of aggregates with distinctive morphologies. (TIFF 226 kb)

## Abbreviations

AD: Alzheimer’s Disease
CNS: Central nervous system
FTDP-17: Frontotemporal dementia with parkinsonism linked to chromosome 17, an autosomal, dominant neurodegenerative disorder caused by mutations in tau gene, including P301L mutation
NFT: Neurofibrillary tangles, deposits of aggregated tau protein in neurons mostly consisting of phosphorylated tau. GFT gliofibrillary tangles, deposits of tau protein formed in glial cells rather than neurons.
TgTauP301L: transgenic mice expressing longest form of human tau protein with P301L mutation
WT: Wild type, non-transgenic littermates of transgenic animals used in the project
MAPT: Microtubule associated protein tau, full name of the tau protein
RD: tau repeat domain present in tau protein
YFP: Yellow fluorescent protein
Chr: Chromosome
