# Supplementary material for: The CNS in inbred transgenic models of 4-repeat Tauopathy develops consistent tau seeding capacity yet focal and diverse patterns of protein deposition
Source: Mol Neurodegener. 2017 Oct 4;12:72. doi: 10.1186/s13024-017-0215-7 (PMC5628424; doi:10.1186/s13024-017-0215-7)
Supplement: Supplementary file 1 — Single Nucleotide Polymorphism (SNP) profiling of congenic and incipient congenic lines. (DOCX 11 kb) [file 13024_2017_215_MOESM1_ESM.docx]

**Table S1. Single Nucleotide Polymorphism (SNP) profiling of congenic and incipient congenic lines.**

| Derivative line | Backcrosses  (gender) | # SNP assays | Call Rate (%) | % match to reference | | |
| --- | --- | --- | --- | --- | --- | --- |
|  |  |  |  | Overall | Full | Half |
| 129/SvEvTac | 12 (M) | 384 | 98.2 | 99.2^a^ | 98.4 | 1.6 |
| FVBN/J | 11 (F) | 384 | 98.7 | 99.9^b^ | 99.7 | 0.3 |
| C57BL/6Tac | 8 (M) | 384 | 99.7 | 98.6^c^ | 99.2 | 0.8 |

^a^distinctions from reference genome located at positions chr 01-26, chr 07-05, 7-19 and chr 14-05; ^b^distinctions from reference genome located at position chr 01-26; ^c^distinctions from reference genome located at positions chr 01-05, chr 05-19, and 14-03. "Full" indicates a homozygous match to reference, "half", a heterozygous match to reference.
