# Supplementary figures and images for: The CNS in inbred transgenic models of 4-repeat Tauopathy develops consistent tau seeding capacity yet focal and diverse patterns of protein deposition
Source: Mol Neurodegener. 2017 Oct 4;12:72. doi: 10.1186/s13024-017-0215-7 (PMC5628424; doi:10.1186/s13024-017-0215-7)

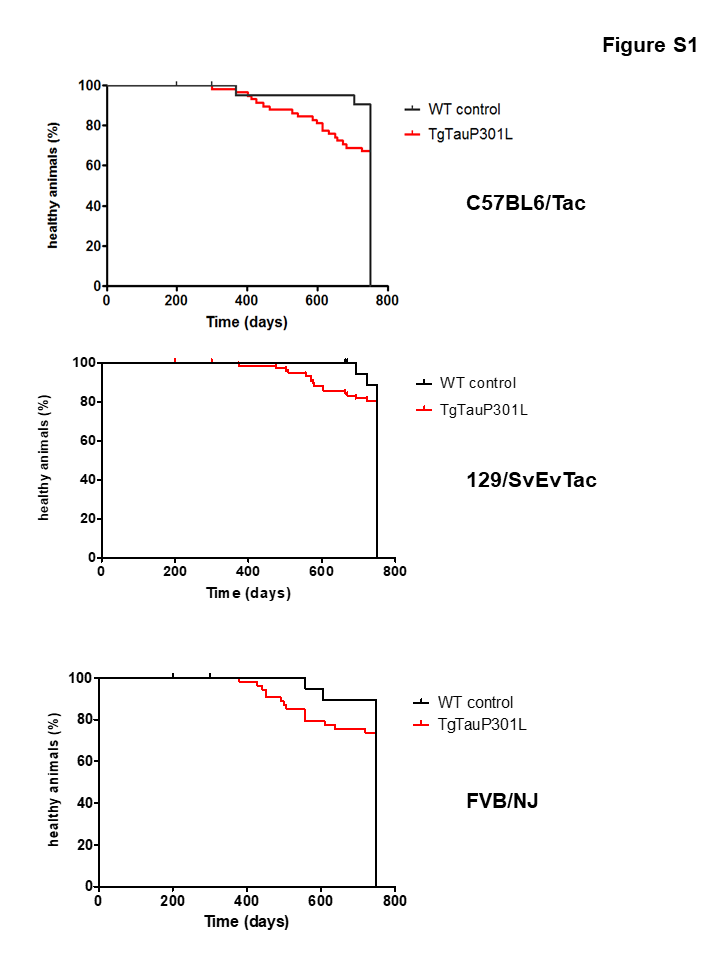

Supplement: Supplementary file 2 — Appearance of disease-associated symptoms in TgTauP301L mice three genetic backgrounds. Time points when disease-associated symptoms were apparent in animals are presented alongside the performance of non-Tg littermates. Survival curves of non-Tg littermates of Tg animals are represented as well. The symptoms recorded for Tg animals are specific for them and none of the non-Tg littermates manifest such symptoms. Non-Tg animal live up to ca. 750 days, however a few animals succumb to natural deaths or have health problems (such as dermatitis or eye infection), requiring euthanasia to be performed. The number of cohort sizes for each group (n) are represented within each graph. All sample groups were terminated at 750 days. (TIFF 83 kb) [file 13024_2017_215_MOESM2_ESM.tif]

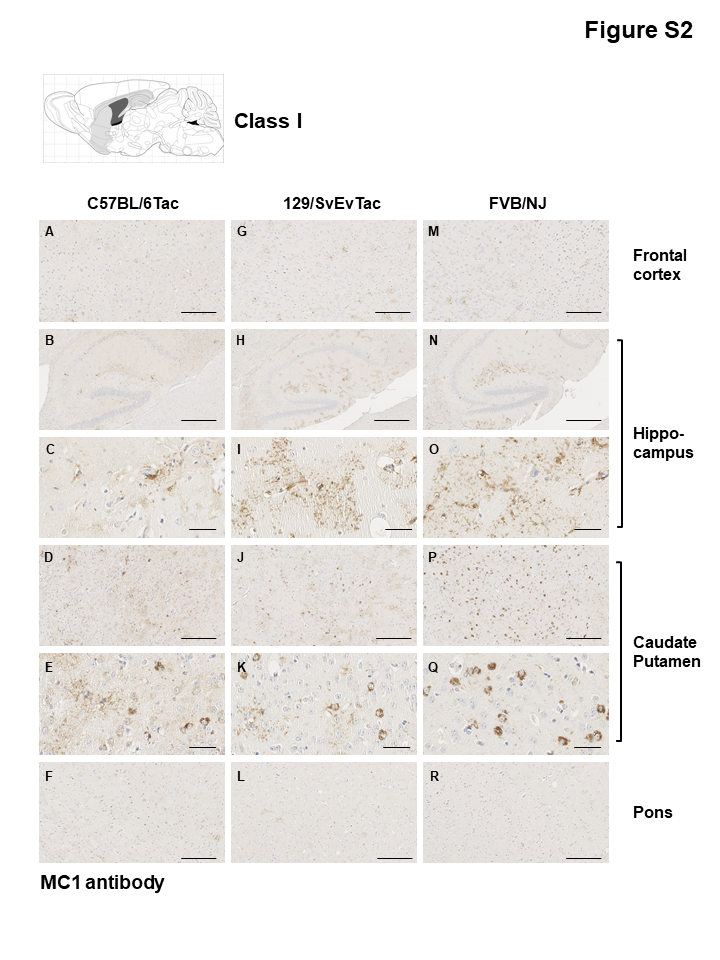

Supplement: Supplementary file 3 — Class I mice. These figures represent the counterparts of Fig. 4. stained with MC1, CP27, RZ3 and PHF1 antibodies, respectively. (ZIP 2909 kb) [file 13024_2017_215_MOESM3_ESM.zip › Supp2.TIF]

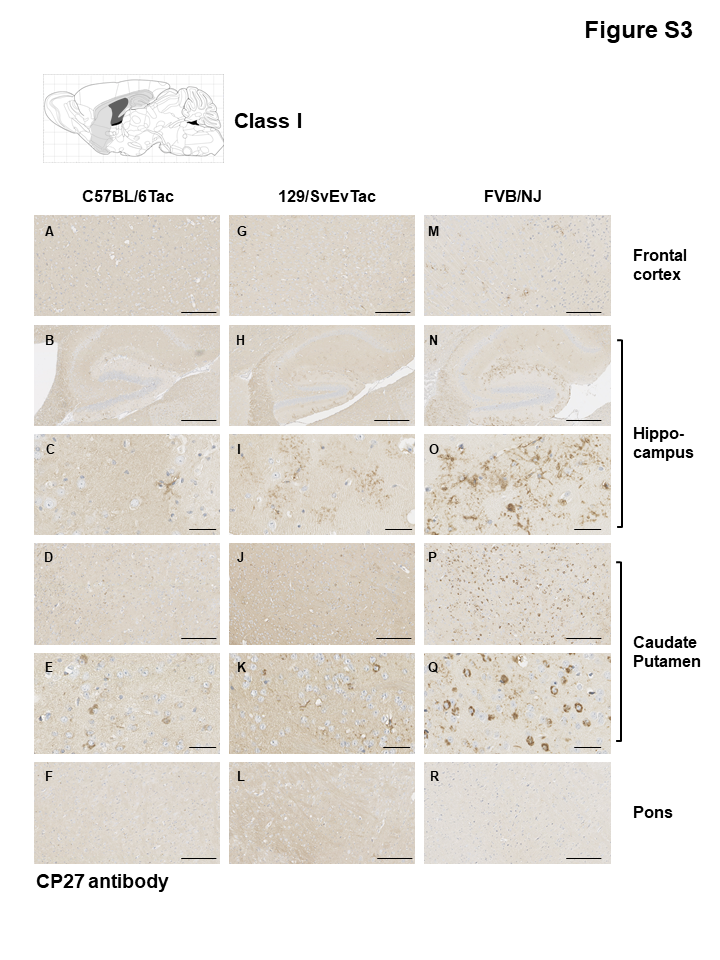

Supplement: Supplementary file 3 — Class I mice. These figures represent the counterparts of Fig. 4. stained with MC1, CP27, RZ3 and PHF1 antibodies, respectively. (ZIP 2909 kb) [file 13024_2017_215_MOESM3_ESM.zip › Supp3.TIF]

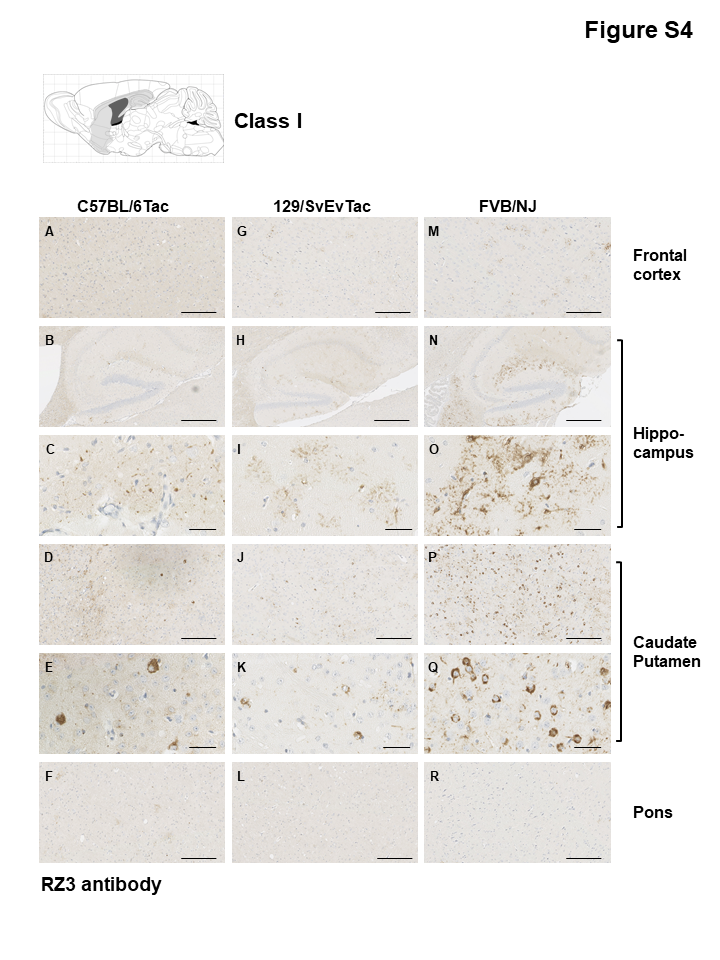

Supplement: Supplementary file 3 — Class I mice. These figures represent the counterparts of Fig. 4. stained with MC1, CP27, RZ3 and PHF1 antibodies, respectively. (ZIP 2909 kb) [file 13024_2017_215_MOESM3_ESM.zip › Supp4.TIF]

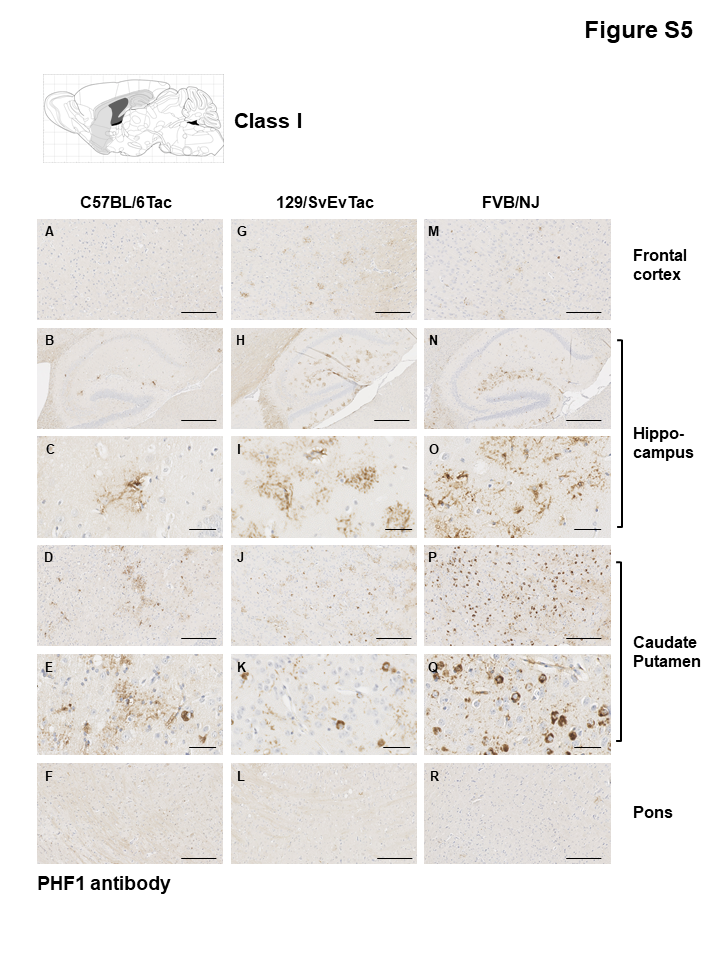

Supplement: Supplementary file 3 — Class I mice. These figures represent the counterparts of Fig. 4. stained with MC1, CP27, RZ3 and PHF1 antibodies, respectively. (ZIP 2909 kb) [file 13024_2017_215_MOESM3_ESM.zip › Supp5.TIF]

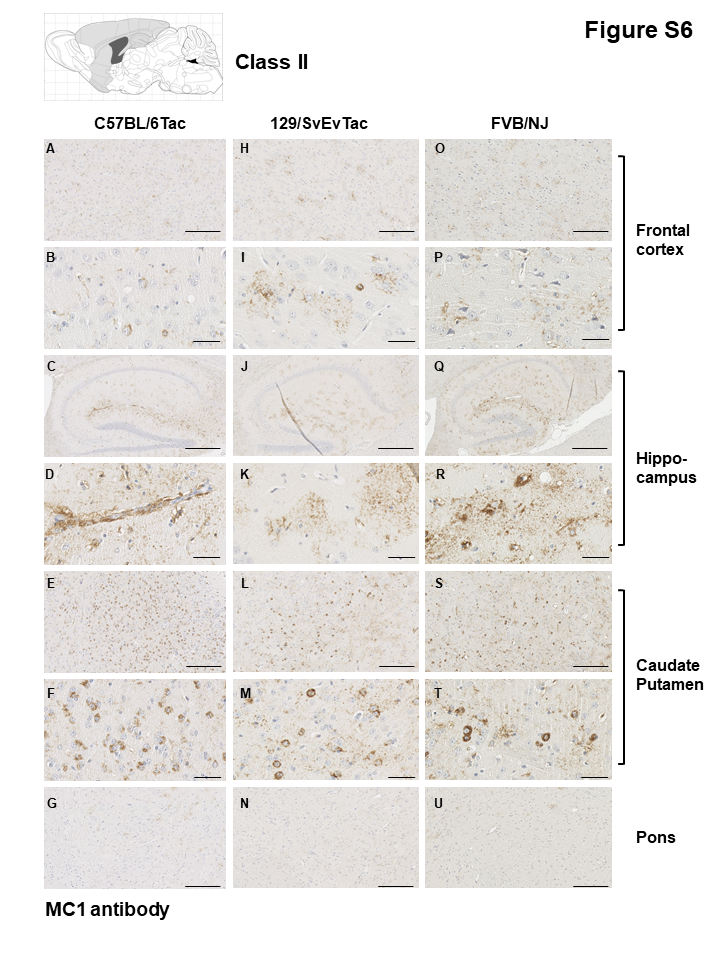

Supplement: Supplementary file 4 — Class II mice. These figures represent the counterparts of Fig. 5. stained with MC1, CP27, RZ3 and PHF1 antibodies, respectively. (ZIP 3613 kb) [file 13024_2017_215_MOESM4_ESM.zip › Supp6.TIF]

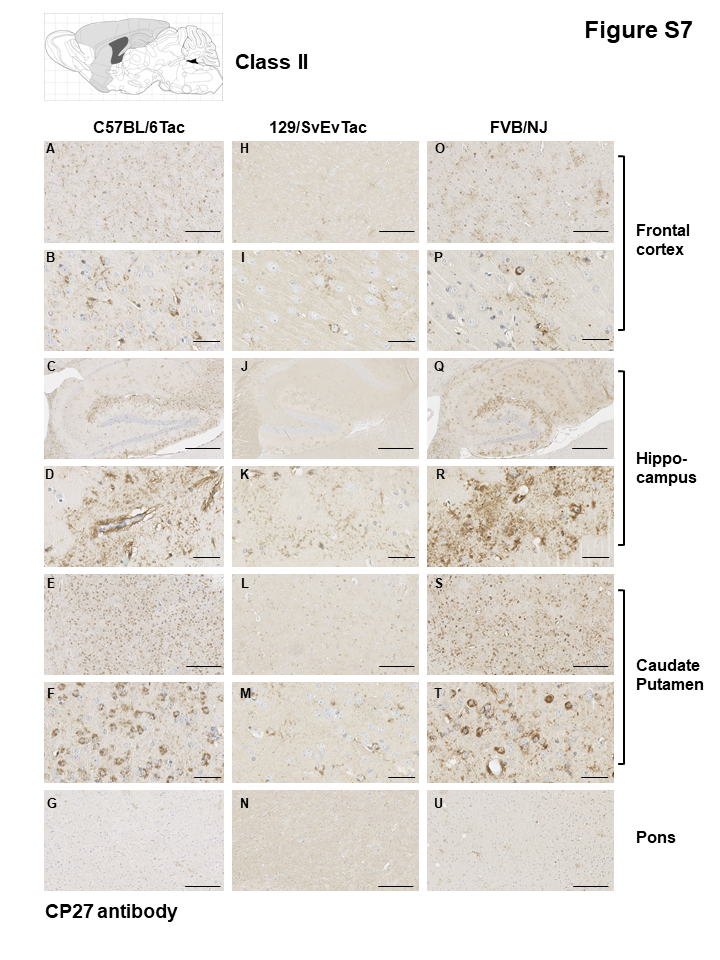

Supplement: Supplementary file 4 — Class II mice. These figures represent the counterparts of Fig. 5. stained with MC1, CP27, RZ3 and PHF1 antibodies, respectively. (ZIP 3613 kb) [file 13024_2017_215_MOESM4_ESM.zip › Supp7.TIF]

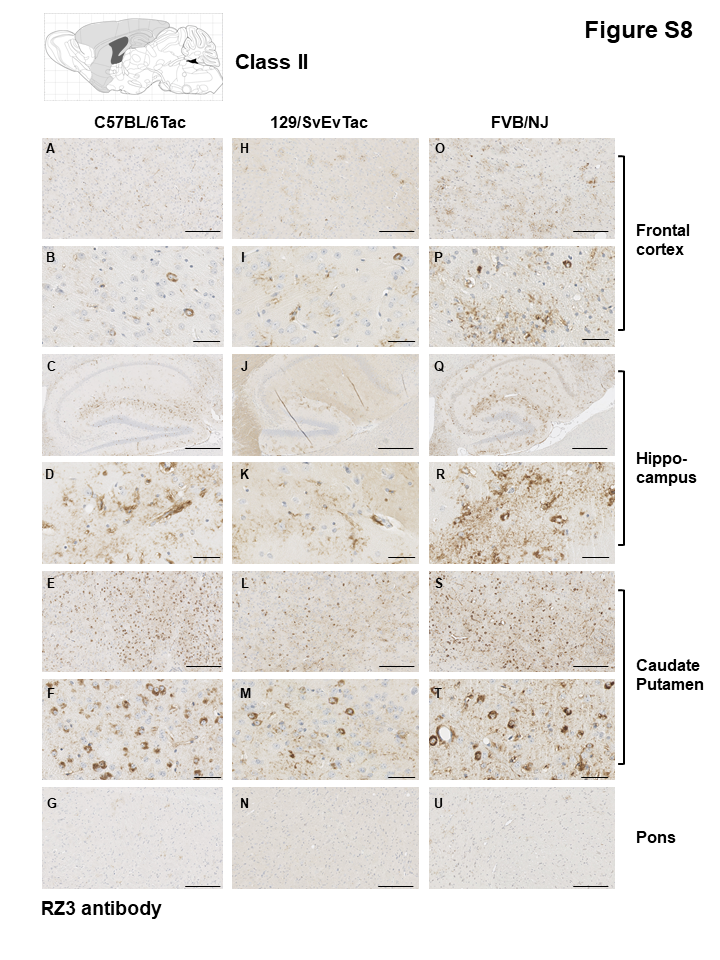

Supplement: Supplementary file 4 — Class II mice. These figures represent the counterparts of Fig. 5. stained with MC1, CP27, RZ3 and PHF1 antibodies, respectively. (ZIP 3613 kb) [file 13024_2017_215_MOESM4_ESM.zip › Supp8.TIF]

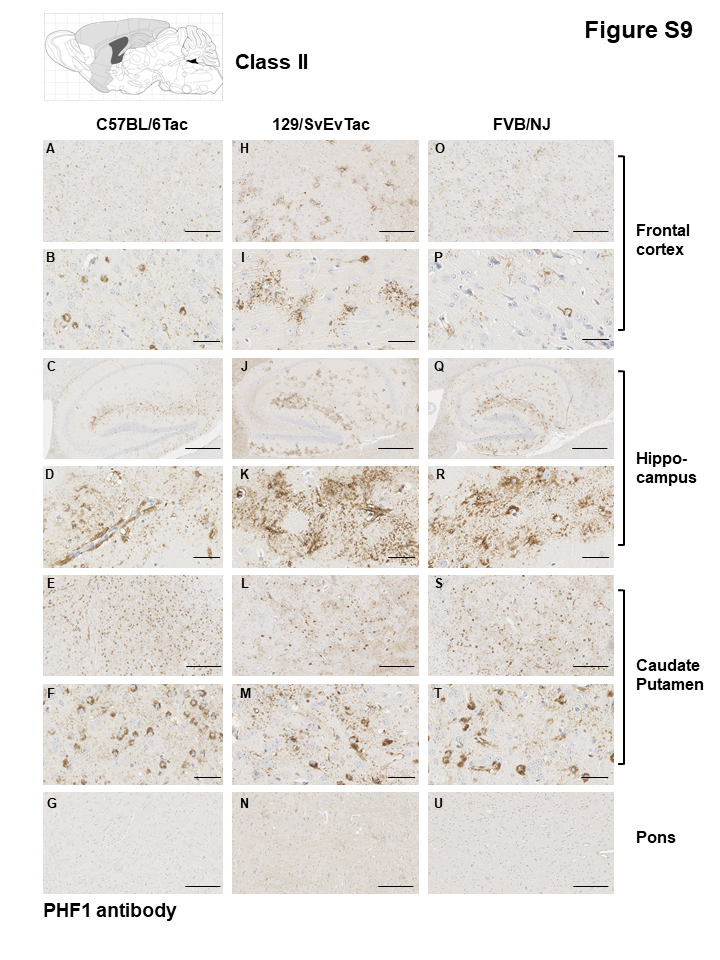

Supplement: Supplementary file 4 — Class II mice. These figures represent the counterparts of Fig. 5. stained with MC1, CP27, RZ3 and PHF1 antibodies, respectively. (ZIP 3613 kb) [file 13024_2017_215_MOESM4_ESM.zip › Supp9.TIF]

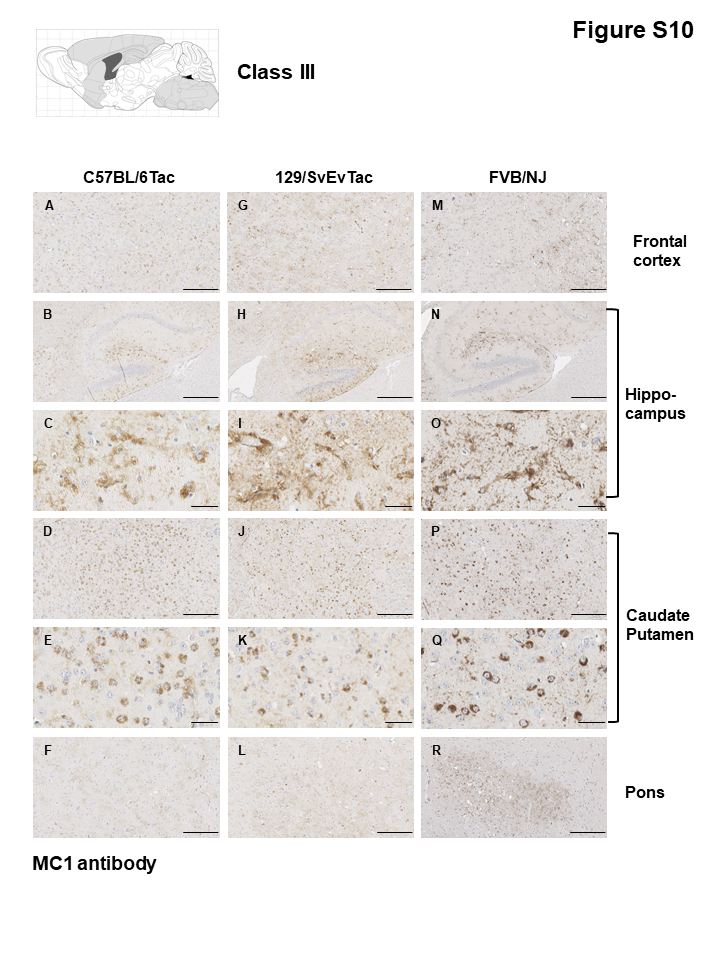

Supplement: Supplementary file 5 — Class III mice. These figures represent the counterparts of Fig. 6. stained with MC1, CP27, RZ3 and PHF1 antibodies, respectively. (ZIP 3238 kb) [file 13024_2017_215_MOESM5_ESM.zip › Supp10.TIF]

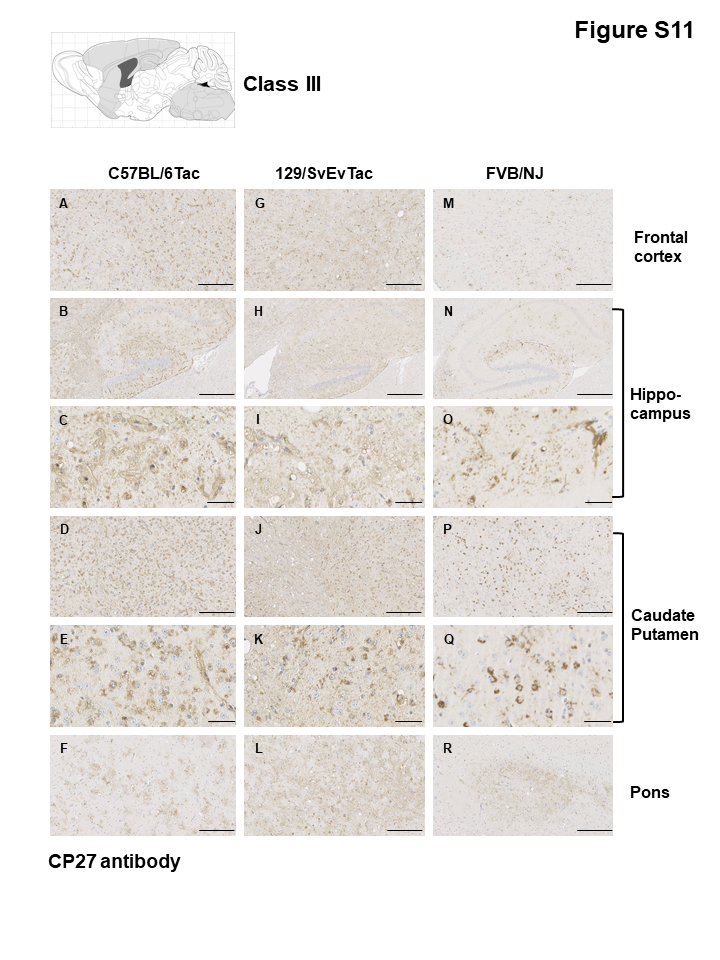

Supplement: Supplementary file 5 — Class III mice. These figures represent the counterparts of Fig. 6. stained with MC1, CP27, RZ3 and PHF1 antibodies, respectively. (ZIP 3238 kb) [file 13024_2017_215_MOESM5_ESM.zip › Supp11.TIF]

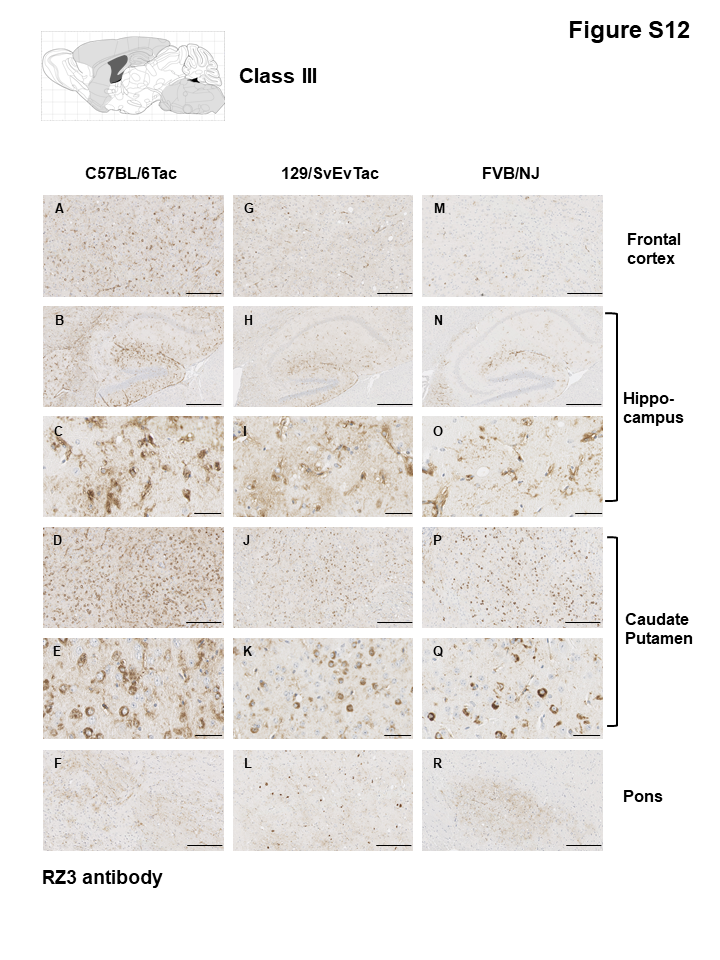

Supplement: Supplementary file 5 — Class III mice. These figures represent the counterparts of Fig. 6. stained with MC1, CP27, RZ3 and PHF1 antibodies, respectively. (ZIP 3238 kb) [file 13024_2017_215_MOESM5_ESM.zip › Supp12.TIF]

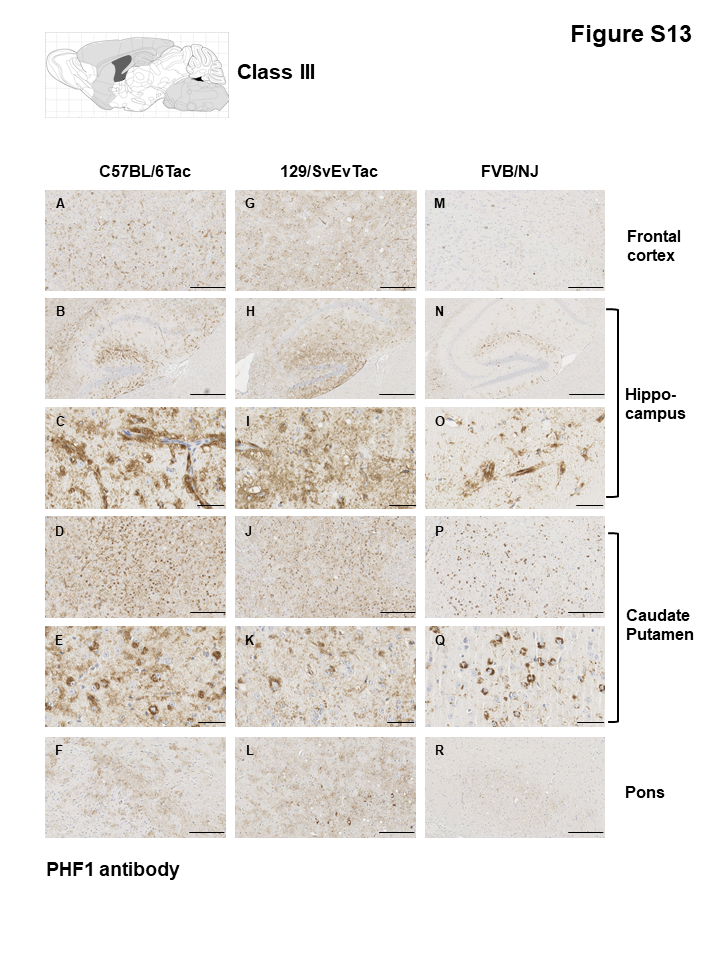

Supplement: Supplementary file 5 — Class III mice. These figures represent the counterparts of Fig. 6. stained with MC1, CP27, RZ3 and PHF1 antibodies, respectively. (ZIP 3238 kb) [file 13024_2017_215_MOESM5_ESM.zip › Supp13.TIF]

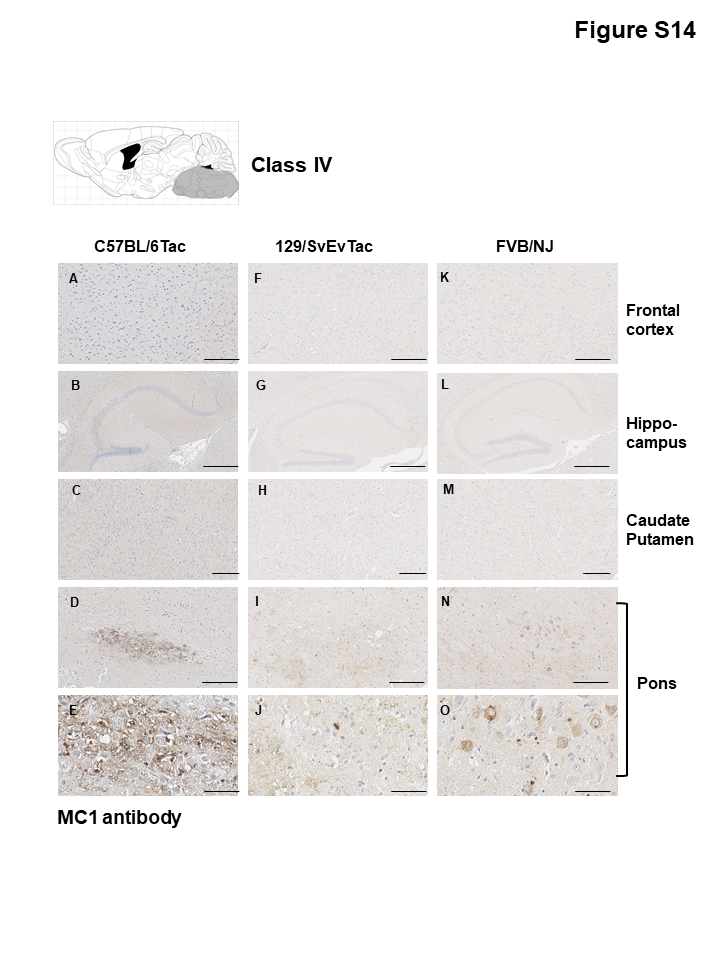

Supplement: Supplementary file 6 — Class IV mice. These figures represent the counterparts of Fig. 7. stained with MC1, CP27, RZ3 and PHF1 antibodies, respectively. (ZIP 2280 kb) [file 13024_2017_215_MOESM6_ESM.zip › Supp14.TIF]

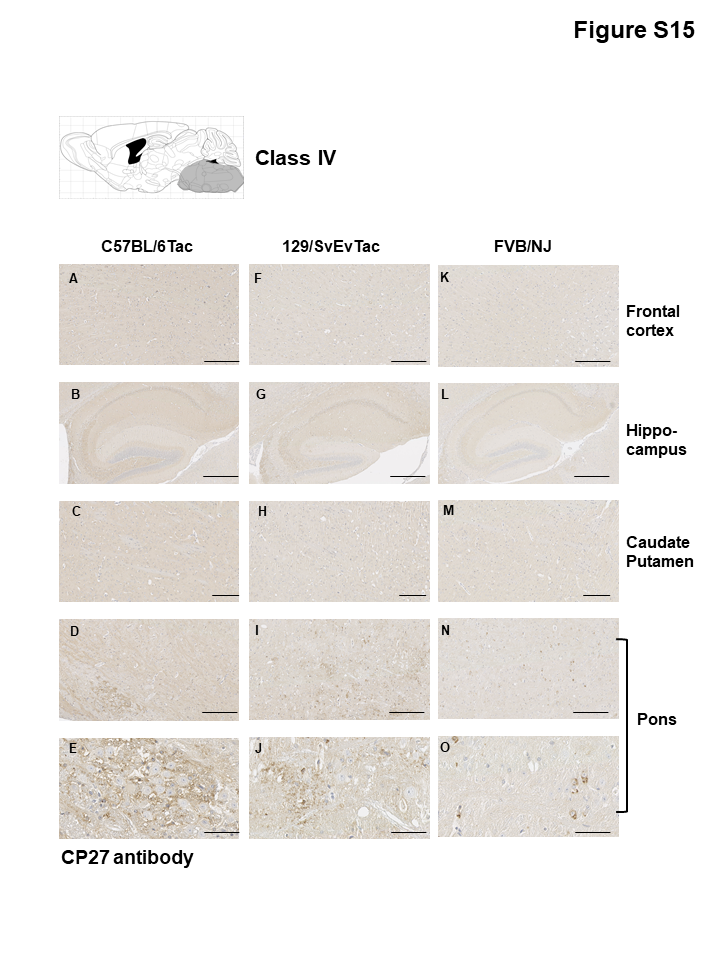

Supplement: Supplementary file 6 — Class IV mice. These figures represent the counterparts of Fig. 7. stained with MC1, CP27, RZ3 and PHF1 antibodies, respectively. (ZIP 2280 kb) [file 13024_2017_215_MOESM6_ESM.zip › Supp15.TIF]

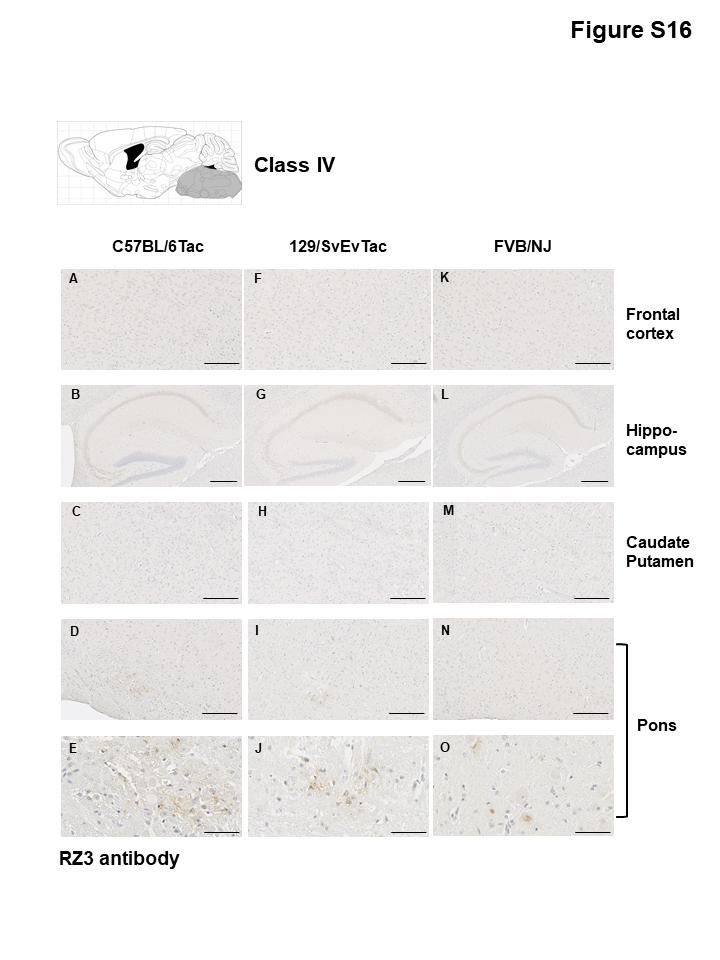

Supplement: Supplementary file 6 — Class IV mice. These figures represent the counterparts of Fig. 7. stained with MC1, CP27, RZ3 and PHF1 antibodies, respectively. (ZIP 2280 kb) [file 13024_2017_215_MOESM6_ESM.zip › Supp16.TIF]

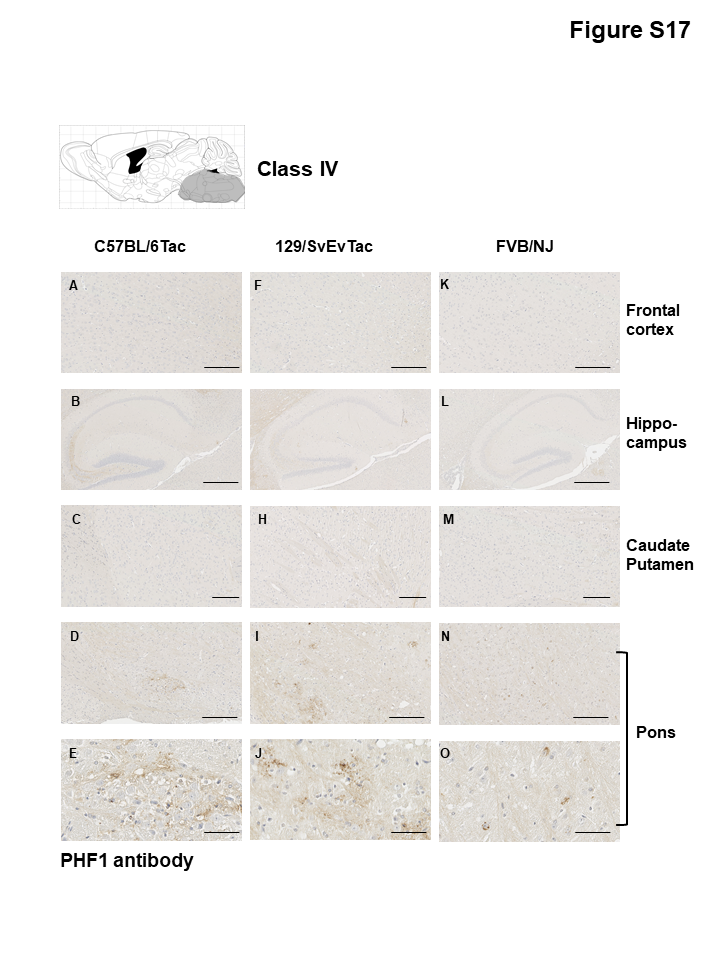

Supplement: Supplementary file 6 — Class IV mice. These figures represent the counterparts of Fig. 7. stained with MC1, CP27, RZ3 and PHF1 antibodies, respectively. (ZIP 2280 kb) [file 13024_2017_215_MOESM6_ESM.zip › Supp17.TIF]

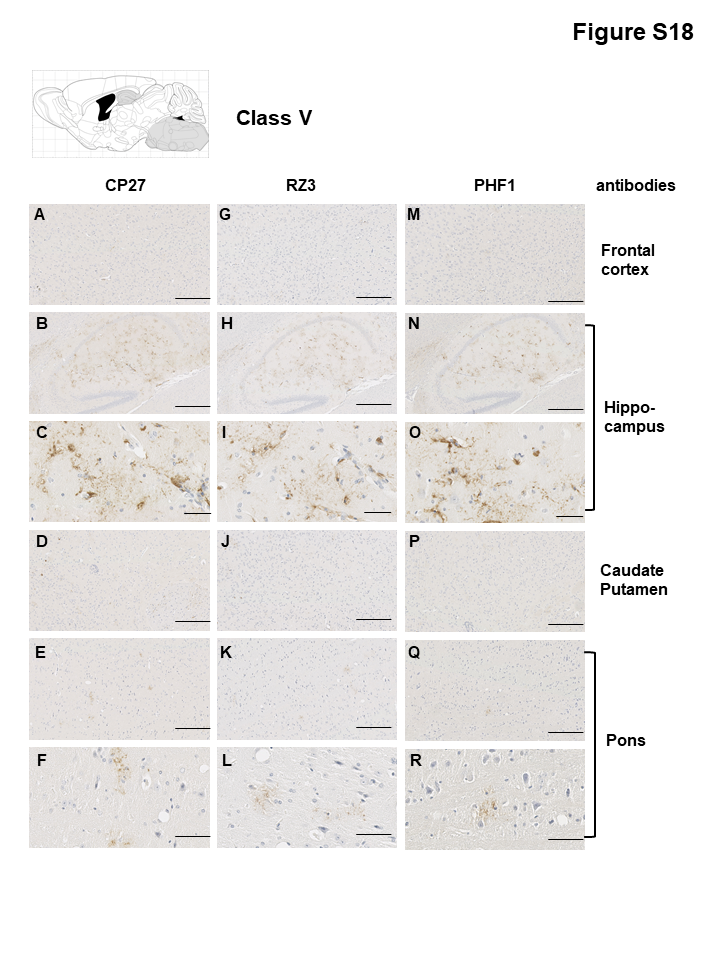

Supplement: Supplementary file 7 — Class V mice. This figure represents a counterpart of Fig. 8 stained with CP27, RZ3 and PHF1 antibodies. (TIFF 690 kb) [file 13024_2017_215_MOESM7_ESM.tif]

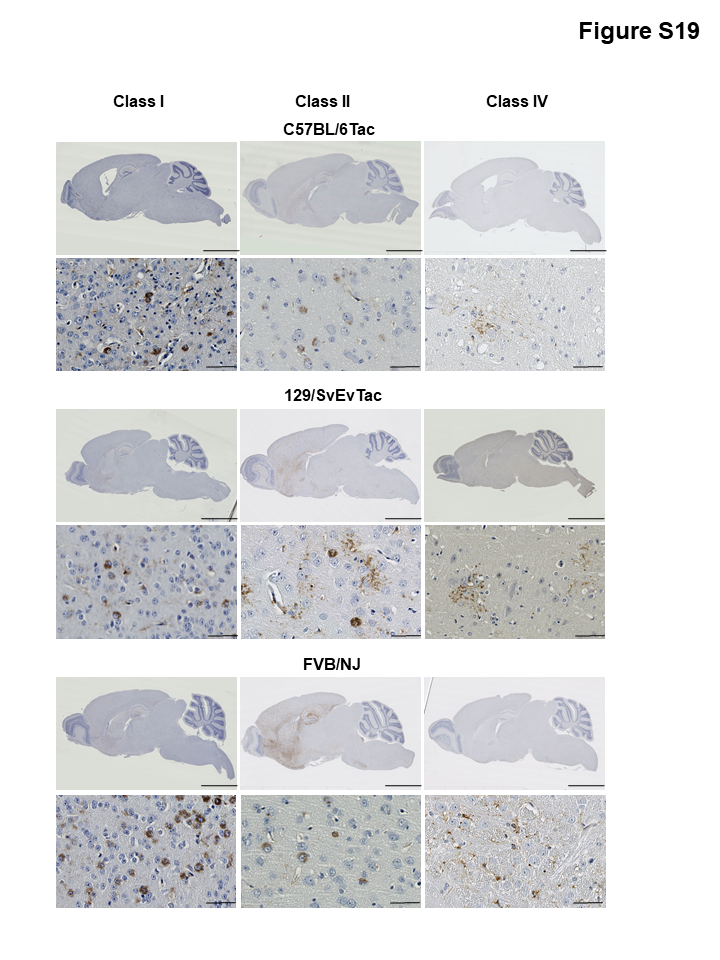

Supplement: Supplementary file 8 — Pathology in aged Tg mice assessed for insoluble Tau species. These data represent the counterparts (other hemi-brains) of the animals assessed for insoluble Tau species in Fig. 12; pathology class and genetic background are annotated. Scale bars for low power views = 2.5 mm, high power views = 50 μm. (TIFF 741 kb) [file 13024_2017_215_MOESM8_ESM.tif]

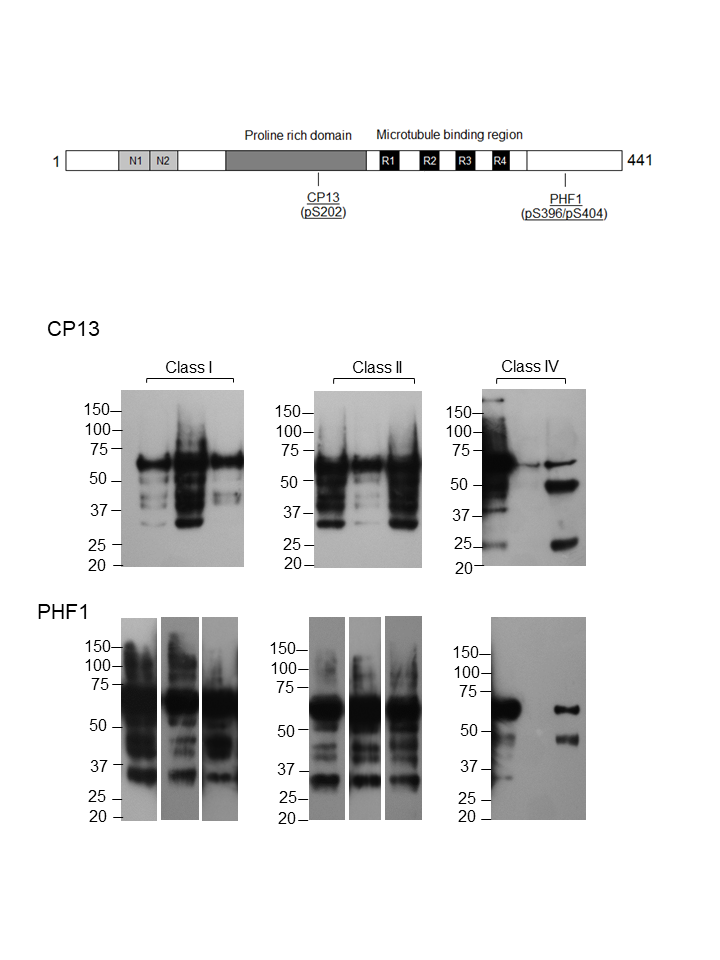

Supplement: Supplementary file 9 — Undigested P3 fraction assessed with CP13 and PHF1 antibodies. A schematic of antibody epitopes is presented. Blot represents P3 fraction from 3 animals of classes I, II and IV. Class I mice at ages 587, 662, and 646 days left to right, class II animals at ages 735, 592, and 658 days left to right, and class IV mice at ages 530, 466, and 639 days left to right. For both blots, 5 μg of total protein was loaded on the gel. Antibody: CP13 (1/500) and PHF1 (1/500). (TIFF 199 kb) [file 13024_2017_215_MOESM9_ESM.tif]

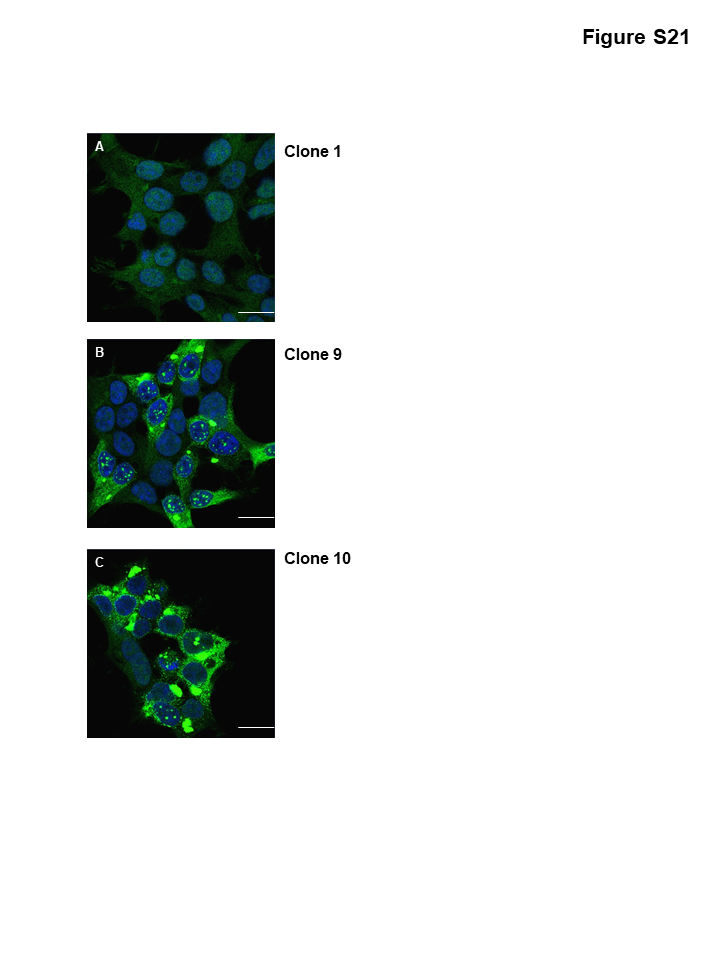

Supplement: Supplementary file 10 — Clones used for fluorescence microscopy assays. Supplement (A-C). Clone 1 (negative control) lysate never seeds inclusions, whereas Clone 9 and Clone 10 seed the formation of aggregates with distinctive morphologies. (TIFF 226 kb) [file 13024_2017_215_MOESM10_ESM.tif]
